# Unprecedented robustness of physics-informed atomic energy models at and beyond room temperature

**DOI:** 10.1038/s42004-026-01965-0

**Published:** 2026-03-31

**Authors:** Bienfait Kabuyaya Isamura, Olivia Aten, Mohamadhosein Nosratjoo, Paul Lode Albert Popelier

**Affiliations:** Department of Chemistry, The University of Manchester, Manchester, UK

**Keywords:** Computational chemistry, Molecular dynamics, Method development, Quantum chemistry

## Abstract

Machine-learned potentials (MLPs) have become widely adopted alternatives to traditional electronic structure and molecular mechanics methods. However, despite excelling on fixed test sets, most MLPs remain prone to instability when deployed in molecular dynamics simulations, particularly at elevated temperatures. Here, we present the first physics-informed Gaussian process (GP)-based atomic energy models that achieve practically unlimited stability in NVT simulations at temperatures as high as 1000 K. Our findings highlight the importance of the GP prior mean function and demonstrate the models’ ability to predict restoring forces that preserve the system’s physical integrity. The quantum chemical topology information embedded in these models acts as an inductive bias to mitigate arbitrary fluctuations in the predicted atomic energies. Finally, the models’ robustness is evidenced by 50 successful simulations of four flexible organic molecules (peptide-capped glycine and serine, malondialdehyde, and aspirin), yielding a cumulative simulation time of 0.5 microseconds completed within two CPU days.

## Abstract (web summary)

Machine-learned potentials (MLPs) have become widely adopted alternatives to traditional electronic structure and molecular mechanics methods; however, most MLPs remain prone to instability when deployed in molecular dynamics simulations, particularly at elevated temperatures. Here, the authors present physics-informed Gaussian process (GP)-based atomic energy models that achieve enhanced stability in NVT simulations at temperatures as high as 1000 K, demonstrating their robustness in simulations of flexible organic molecules (peptide-capped glycine and serine, malondialdehyde, and aspirin) with a cumulative simulation time of 0.5 microseconds completed within two CPU days.

## Introduction

Machine-learned potentials (MLPs) were introduced about two decades ago, aspiring to bridge the gap between expensive electronic structure methods and approximate molecular mechanics methods^1–3^. Their design has since been considerably refined, leading to a variety of molecular descriptors, learning frameworks, and application domains^4–10^. Training a single-molecule MLP involves establishing a mapping between a given representation of the system and some target physical properties, usually either energies or atomic forces, or both. The quality of a trained MLP is generally assessed by testing its predictive accuracy on out-of-sample geometries. Most state-of-the-art MLPs have unambiguously demonstrated their ability to achieve miniscule mean absolute errors (MAEs) on fixed test sets (taken from an existing dataset that also provided the training set). However, these static MAEs can be misleading. The reason is twofold: (i) MAEs can vary by orders of magnitude depending on the nature of the training and test sets^11^, (ii) they do not always reflect the expected robustness of the underlying models in molecular dynamics (MD) simulations^12^.

Over the past decade, several studies have shed light on the origin of the precarious robustness of mainstream MLPs. These investigations have highlighted how crucial it is to train MLPs on datasets that faithfully represent all the key topological aspects of the target potential energy surface (PES). For instance, it was revealed that having conformational holes in the training set increases not only the models’ vulnerability to extrapolation^13^, but also the likelihood of making erroneous predictions of energies and atomic forces during MD simulations. Besides the representativeness of the training set, another key factor to consider when designing a robust MLP is the expressiveness of the molecular descriptor. Ideally, a good descriptor should capture both short-range and long-range interactions between atoms^14^, while also complying with the translation, rotation, and permutation symmetries of the target property^15^. Unfortunately, even when both the dataset and the descriptor seem appropriate, it is surprisingly common to see MLP-driven simulations fail^16^ against all expectations. While most state-of-the-art MLPs can survive mild thermal agitation at very low temperatures, their robustness tends to quickly decline at elevated temperatures, where more extensive configurational changes and transitions between metastable states are likely to occur^16^. Due to the finite amount of knowledge that can be embedded in any model, MLPs are inherently at risk of extrapolation, which carries a non-zero risk of simulation failure. For any non-reactive force field, such as our in-house physics-informed FFLUX force field^17,18^, how long a data-driven simulation remains stable depends on the underlying model’s extrapolation capability and its ability to predict the restoring forces necessary to counteract any explosion or implosion of the system^19^.

In this work, we report the first-ever physics-informed Gaussian process (GP)-based atomic energy models that produce stable multi-nanosecond NVT simulations at temperatures as high as 1000 K. Popular Behler-Parrinello-like MLPs have site energies that are not only determined by the learning architecture, but that can also vary in arbitrary ways and have no physical meaning. Unlike those MLPs, the models reported here are trained on pre-computed and interpretable atomic energies whose values emerge from rigorous quantum mechanical laws^20^. We illustrate our approach on four systems, including peptide-capped glycine (GLY), peptide-capped serine (SER), malondialdehyde (MAL), and aspirin (ASP). Chosen for their structural flexibility, both these and related systems have posed significant challenges to numerous MLPs in past attempts to achieve stable MD simulations^12,21^. Our experiments highlight the crucial role of GPs’ prior mean function *m*, and indicate that shifting *m* toward high atomic energy states produces exceptionally robust models that lead to stable simulations at and beyond room temperature. We attribute the exceptional robustness of our GP-based atomic energy models to their inherent capability to indirectly predict restoring forces, which allow them to prevent bond explosions and implosions. Finally, using our 1000-point models, we managed to propagate 50 independent MD simulations for 10 ns each, reaching an aggregated simulation time of 0.5 microseconds at 500 K.

## Results

### Static performance of atomic IQA energy models

GPR models were trained on physics-informed atomic energies computed following the Interacting Quantum Atoms (IQA) formalism^20^ using 1000 training geometries generated through well-tempered metadynamics (WTMetaD)^22^. The notation MFn (with n $\in \left\{{\mathrm{1,2,3,4,5}}\right\}$ and MF standing for mean function) is used throughout this work to denote the different prior mean functions defined in the Methods section. Alternatively, we also use the shorthand SYSTEM-1000-MFn to indicate a 1000-point model of a given SYSTEM trained using the MFn mean function. A detailed discussion of the quality of the reference atomic energies and the structural diversity of our datasets, including a comparison with two other samplers, is provided in Section 1 of the Supporting Information (SI). The data in Table S1 illustrates the excellent quality of our reference datasets, with more than 99.8% of molecular IQA energies lying within 1 kcal/mol from the reference DFT energies. According to Figures S1 and S2, the WTMetaD sampler achieves a satisfactory coverage of the target conformational spaces, outperforming both the classical^23^ and the unbiased semi-empirical^24^ samplers.

**Fig. 1:**
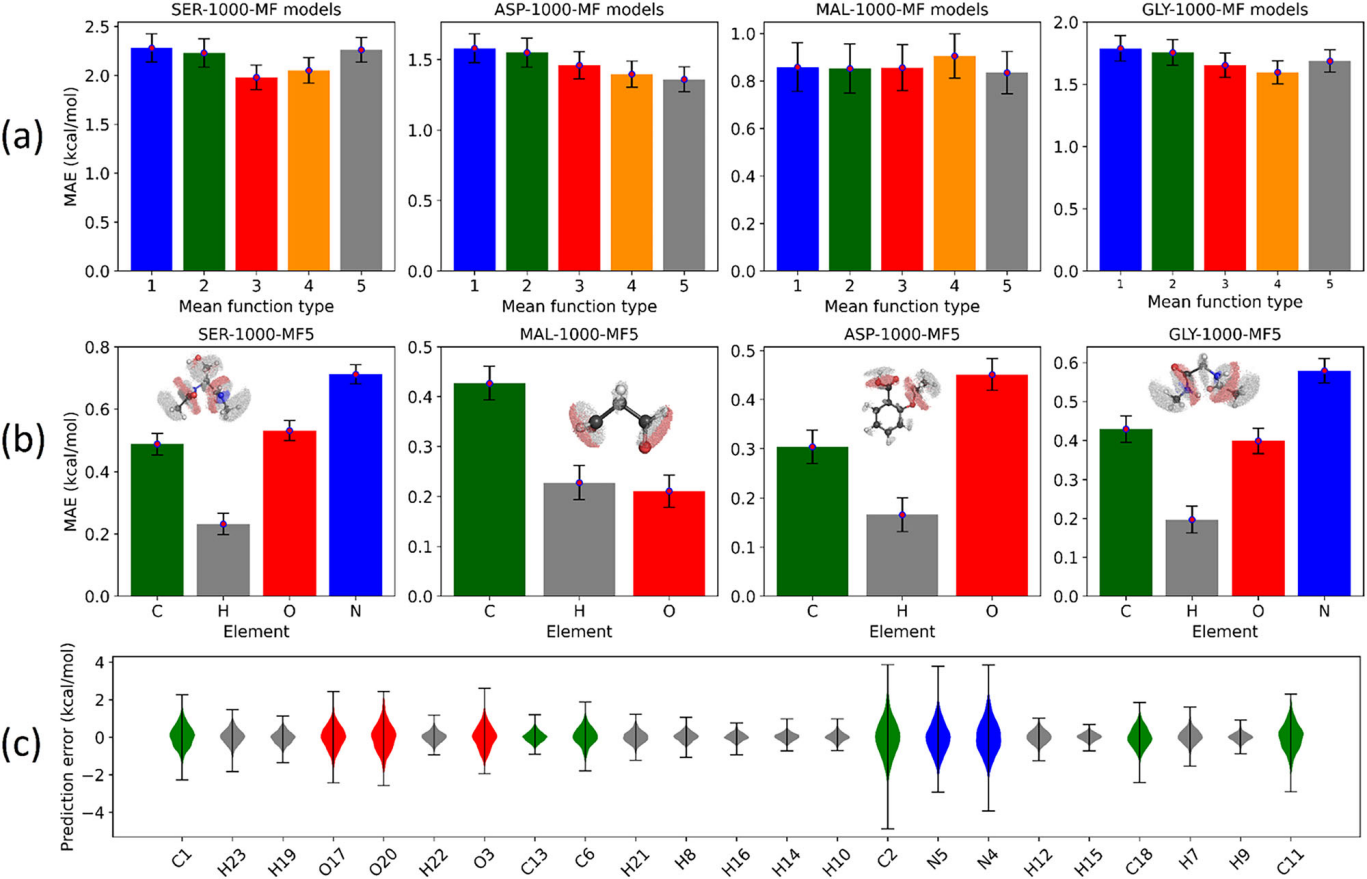
Static performance of GP-based atomic energy models. All the models were trained on 1000 geometries and tested on an out-of-sample test set of 1000 geometries. These models only differ by the definition of the prior mean function, which is MF1, MF2, MF3, MF4 and MF5 as respectively defined in Eqs. 7–11. **a** compares the molecular MAEs of various models of SER, ASP, MAL and GLY. **b** gives element-wise MAEs of models trained using MF5. **c** gives atomic MAEs for the SER-1000-MF5 model. Notice that heavy atoms have larger errors than hydrogen atoms. The labelling of serine atoms follows the same order as in the xyz files deposited as part of the SI. Accordingly, in this serine, C1 is the alpha carbon bonded to three heavy atoms (backbone carbon C2, N5 and beta carbon C18), while C6 and C13 are involved in methyl groups; O20 forms an O-H bond with H23, while O3 and O17 are involved in carbonyl groups. Notice that C2 has the largest error bar, which is consistent with the large fluctuation of its reference atomic energies. The hydrogen atoms involved in N-H (H7 and H21) and O-H (H23) bonds also exhibit larger error bars than their analogs involved in the two methyl groups (H8, H9, H10, and H14, H15, and H16).

Figure 1 illustrates the static performance of our atomic energy models. Not surprisingly, these models achieve reasonable predictive accuracy, comparable with the literature^25^. In particular, all element-wise models achieve MAEs below the familiar threshold of chemical accuracy typically set at 1 kcal mol^-1^. Furthermore, Fig. 1c suggests that “heavy” (i.e., non-hydrogen) atoms exhibit higher atomic energy errors than hydrogen atoms. This finding can be rationalized by considering the distinct range of the target IQA energies, which is larger for heavy atoms. Finally, close inspection of Fig. 1a suggests that the static MAEs are only minimally affected by the choice of prior mean function. Although one may expect the respective models to exhibit similar robustness when deployed in MD simulations, this is not the case. We demonstrate in the next section that the choice of *m* is crucial when it comes to the robustness of GP-based MLPs.

### Simulation stability and robustness tests

**Fig. 2:**
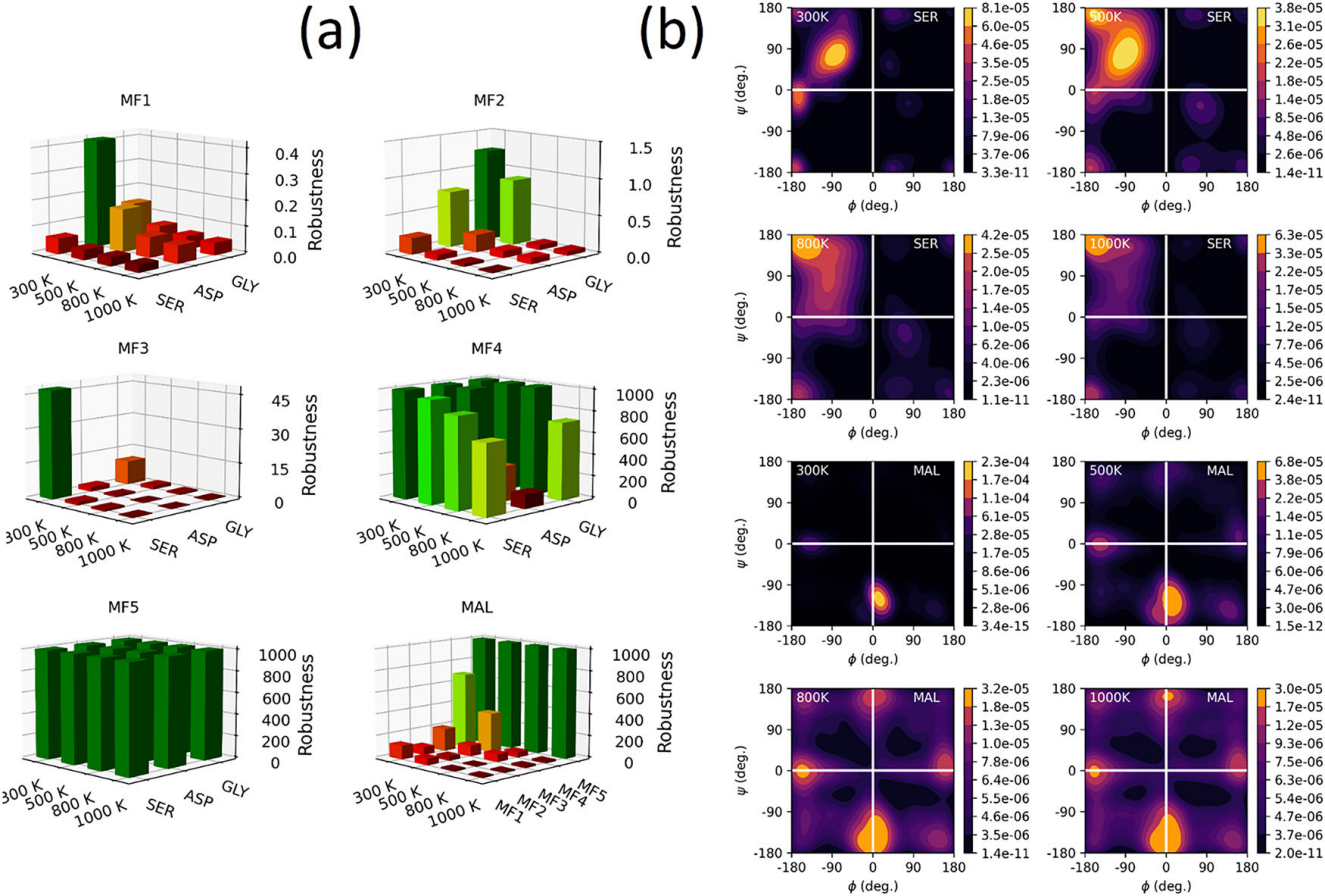
Robustness of GP-based atomic energy models. **a** robustness of GP-based atomic energy models deployed in NVT simulations at 300 K, 500 K, 800 K and 1000 K. The *x* and *y* axes indicate the simulation temperatures and system names. The *z*-axis is the robustness score computed as the average simulation time in ps. The subplots are named according to the various mean functions, except the bottom right plot (MAL), which gives the results for MAL-1000-MFn models. Each bar summarizes 10 MD simulations initiated from structurally diverse geometries. By default, these simulations were set to run for a maximum of 1000 ps, corresponding to 4 million steps (timestep of 0.25 fs). For GLY, SER, and ASP simulations, the average robustness scores over all temperatures and systems were 0.10 ps, 0.32 ps, 5.6 ps, 801.3 ps and 1000.0 ps for MF1, MF2, MF3, MF4 and MF5, respectively. **b** FFLUX MD sampling of SER (top 4 panels) and MAL (4 bottom panels) configuration space at 300 K, 500 K, 800 K, and 1000 K (using MF5-based models). The configuration samples are projected along the directions of the two C-C-C-O dihedral angles for MAL and along the standard ϕ and ψ torsional angles for SER. Each subplot was obtained from a sample of 100,000 geometries made of 10,000 frames, equally collected from each run.

Unlike static performance tests, which can be misleading^21,26^, robustness tests are the ultimate measure of the usefulness of an MLP intended to be deployed in MD simulations. To assess the robustness of our GP-based atomic energy models, we deployed them in NVT simulations at increasingly higher temperatures. Figure 2a illustrates the robustness of SER, ASP, GLY and MAL models. The first and most striking observation in Fig. 2a is the dramatic difference in robustness scores between various series of models. Models based on MF1 and MF2 exhibit extremely poor robustness scores, with the average simulation time failing to exceed 1.5 ps. The only exception is the smallest system (MAL), whose MF2-based simulation remained stable for ∼72 ps at 300 K. The models based on MF3 demonstrate significantly improved robustness compared to the previous ones, reaching robustness scores of up to 45 ps in the case of SER at 300 K. Nevertheless, their performance remains considerably lower than that of MF4 and MF5-based models, whose trajectories are orders of magnitude more stable. In particular, MF5-based models achieve a perfect robustness score of 1000 ps for all four systems and temperatures.

We illustrate the effect of the simulation temperature on the sampling of MAL’s and SER’s configuration spaces in Fig. 2b. Similar results for GLY and ASP are provided in Fig. S3 of the SI. As anticipated, we find that increasing the simulation temperature facilitates the coverage of a broader configuration space. For instance, at 300 K, the unbiased SER simulation is stuck in the top left quadrant of the Ramachandran plot, a region favorable for the formation of beta sheets. Elevating the simulation temperature allows the system to venture into higher energy states. The same observation applies to MAL whose conformational sampling is improved at high temperature. It is interesting to note that, even at 1000 K, FFLUX simulations remained largely within the configurational space spanned by the training data (see Figs. 2 and S2). In the few instances where the trajectories briefly left this domain (and the bond distortion challenges reported in “Mean function and relaxation of high-energy structures" and “Simulation temperature and prediction of restoring forces”), the models relied on their extrapolation capability to preserve physicality by predicting restoring forces.

### Mean function and relaxation of high-energy structures

**Fig. 3:**
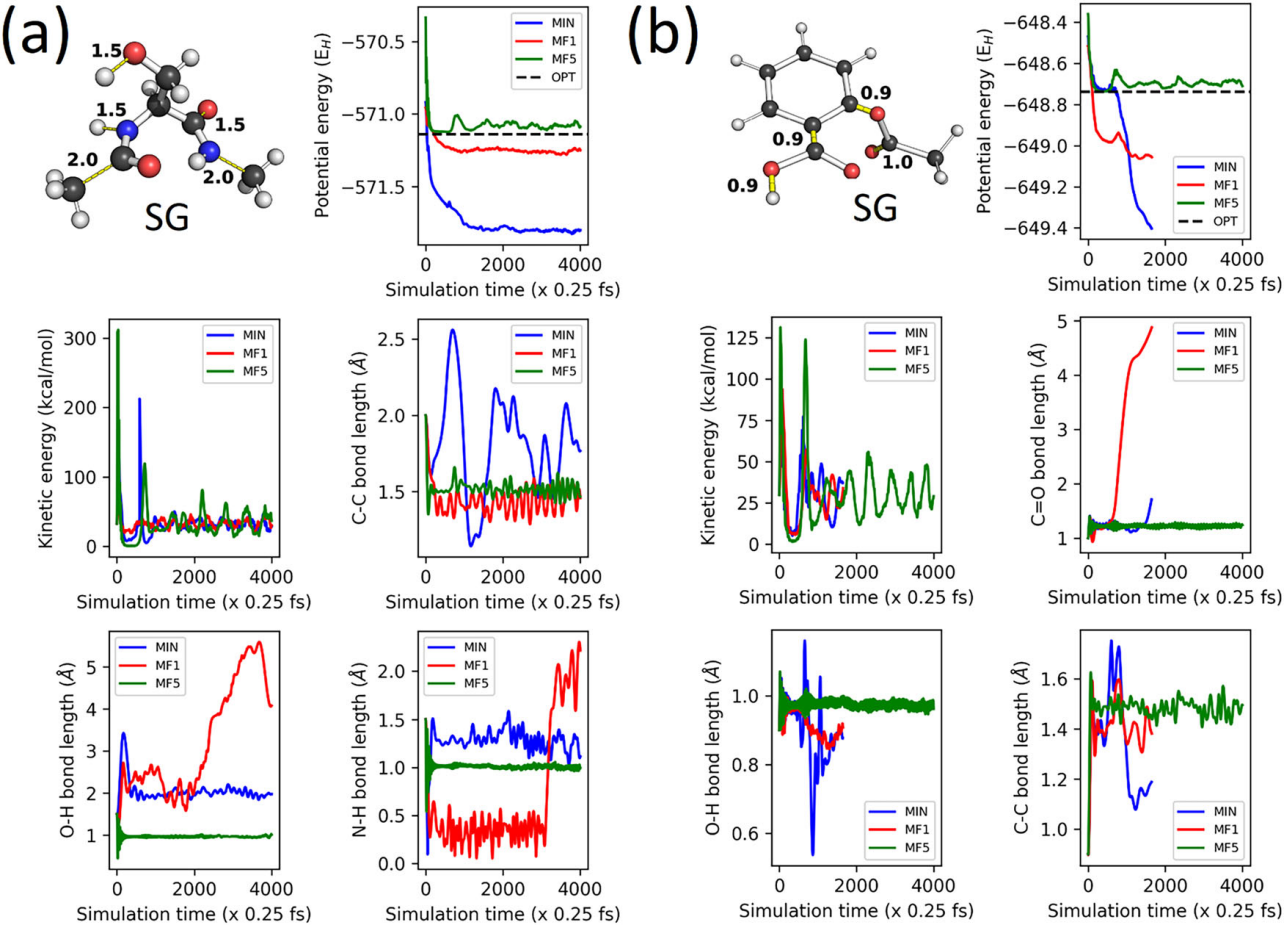
Relaxation of high-energy structures using FFLUX models. First 4000 timesteps (or 1 ps) of a series of GP-based NVT simulations (500 K) of **a** serine and **b** aspirin with high-energy starting geometries (SGs). The models performing these simulations were all trained on 1000 geometries and achieved indistinguishable static MAEs. All the simulations were propagated without explicit equilibration. The plots illustrate the potential and kinetic energy of the system during this 1 ps. They also indicate the evolution of the bonds (in yellow) that were initially perturbed. The two SGs were respectively 227 kcal/mol and 193 kcal/mol higher in energy than the nearest minimum on the respective PESs. The erratic behavior of MIN and MF1-based models causes premature crashes of the corresponding simulations. This is reflected in the evolution of both the potential energy of the system and the selected bonds. The horizontal dashed black lines indicate the potential energy of an optimized structure of the system.

To rationalize the robustness results, we first investigated the extrapolation power of our GP-based models by assessing their ability to recover from high-energy geometries. For this purpose, we performed NVT simulations of SER and ASP at 500 K using high-energy starting geometries (SGs). To emphasize the uniqueness of MF5-based models, we examined three different mean functions, including the MF1 and MF5 defined in Eqs. 7 and 11, respectively, as well as MIN, which is defined as MIN $=\min \left\{{E}_{{IQA}}^{A}\right\}$. The latter function was chosen to investigate how shifting *m* toward even lower-energy states affects the robustness of GP-based atomic energy models. The SG for SER’s simulation (see Fig. 3a) was obtained by manually stretching the O-H bond, one N-H bond, and one C=O bond all to 1.5 Å, and the two terminal C-C and C-N bonds each to 2.0 Å. For ASP, four bonds were compressed from their equilibrium length to 0.9 Å, as shown in Fig. 3b.

According to Fig. 3a, only the MF5-based model successfully recovered from the non-physical SG, stabilized within 1 ps before running for 1 ns without crashing. This model quickly relaxed the O-H, N-H, and terminal C-C bond lengths from initial values of 1.5 Å, 1.5 Å, and 2.0 Å, to the more reasonable lengths of 0.98 Å, 1.0 Å and 1.58 Å, respectively. In contrast, both the MF1 and MIN-based models suffered from dramatic failures during the early stages of the simulation. Their erratic behavior is reflected in the explosion of the molecule and the unusual drop in the system’s potential energy.

The results for ASP were equally alarming for the MF1 and MIN-based models, which prematurely failed before even completing 2000 timesteps. In contrast, the MF5-based model managed to amend the extra-short bonds in the SG and propagate the simulation for 1 ns. Figure 3b shows that this model easily restored the O-H and C–C bonds from the initial values of 0.9 Å to the more reasonable ones of ∼1.0 Å and ∼1.5 Å, respectively, and this within 100 timesteps. We show in Fig. S4 of the SI that the same model also addressed unrealistic angles and dihedrals in the same manner as for compressed bonds.

### Simulation temperature and prediction of restoring forces

To shed more light on the origin of the unprecedented robustness of our MF5-based models, we inspected the predicted atomic forces in the initial stages of a simulation with a very unstable SG. We also examined how these forces were affected by the simulation temperature. Here we only report the results for MAL since qualitatively equivalent results were obtained for SER and ASP, which are more complex than GLY such that GLY need not be analyzed. All the MAL simulations were initiated from the same SG, generated by concomitantly compressing the two C-C bonds to 0.9 Å and stretching the two C=O bonds up to 2.0 Å.

Figure S5a–f illustrates the evolution of the system’s energy and relevant bonds during the most eventful part of the simulation, in this case, the first 1000 fs. Notice that the MAL-1000-MF5 model quickly and irreversibly relaxed the unphysical SG, before leading to a 1-ns stable simulation afterward at all four temperatures. The fact that the SG lay 1149 kcal mol^−1^ higher in energy than the optimized structure of MAL at the B3LYP/6-31 + + G(d,p) level confirms the exceptional ability of this non-reactive model to recover from non-physical structures, thereby preventing the formation of unphysical configurations. We highlight that, regardless of the temperature, all the simulations proceeded in two phases: (a) a very short stabilization/equilibration phase triggered by the model itself to amend unrealistic internal coordinates, and (b) a production phase characterized by more stable statistics.

**Fig. 4:**
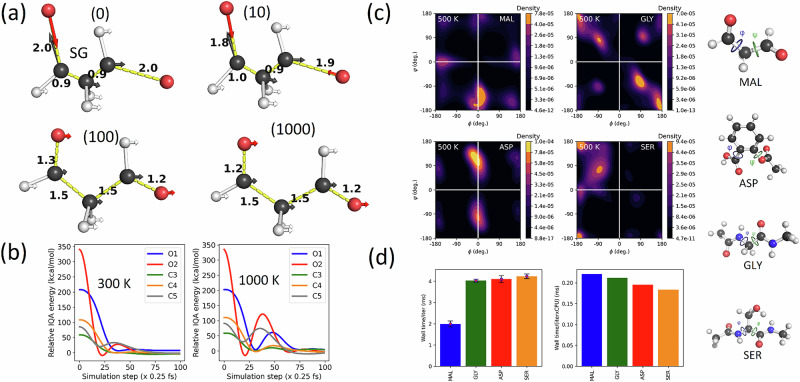
Prediction of restoring forces in distorted malondialdehyde (MAL) structures. **a** Atomic force vectors predicted by the MAL-1000-MF5 model after an increasing number of simulation steps (0, 10, 100 and 1000) at 500 K, initiated from a nonphysical geometry with respective to C-C and C=O bond lengths of 0.90 Å and 2.0 Å. The magnitude of each force vector was scaled between ϵ and 1.0 Hartree/Bohr (ϵ being a small number). The distance (in Å) between relevant bonded atoms is indicated in black. The dashed yellow lines highlight the bonds of interest and ensure that the atomic force vectors are visible when they align with the bond direction; **b** relative IQA energies of the O and C atoms involved in the perturbed bonds (with atomic energies at timestep 4000 chosen as the reference, to emphasize the stabilization occurring in the early stages of the simulation); **c** unbiased 500 K NVT sampling of MAL, GLY, ASP and SER configuration spaces using 1000-point GP-based atomic energy models based on MF5. Each contour plot was generated from a population of 2,000,000 geometries, obtained by sampling 40,000 geometries from each of the 50 runs. The ϕ and ψ dihedral angles are defined in the utmost right column. The color bar indicates the sampling density, where the density increases from black to light orange; **d** the bottom left plot indicates the simulation cost in terms of wall time per MD simulation step for different systems. The number of CPU cores was set to be equal to the number of atoms, as this provides the maximum OpenMP acceleration in our current DL_FFLUX implementation.

Figure 4a depicts the orientation of atomic forces at timesteps 0, 10, 100, and 1000 for the 500 K simulation. The predicted forces at timestep 0 and 10 are directed to stretch the short C–C bonds and compress the long C=O bonds. Within only a hundred time steps, the predicted restoring forces smoothly stabilized the O and C topological atoms involved in the perturbed bonds. This local effect was more pronounced for the O2 atom (Fig. 4b), which witnessed an energy decrease of up to 350 kcal mol^−1^. This consequently produced a proportional minimization of the system’s potential energy. In line with the ongoing relaxation of the system, Fig. S5a, b suggests a sudden early drop in both the total and potential energies. This relaxation period spreads over several simulation timesteps, forming waves that are reminiscent of a dampened harmonic oscillator. The height of the dampening waves in the total energy curves is more pronounced and more spaced at higher temperatures. The same wave-like pattern appears on the maximum atomic force (max-force) curves illustrated in Fig. S5g, which also indicates that max-force decreases rapidly during the first 1000 fs, reaching a minimum value typical of an optimized structure. The position of this minimum depends on the simulation temperature: the higher the temperature, the higher and earlier this minimum is reached.

### Extended MD simulations and deployment cost

The earlier reported robustness tests, based on 10 independent MD simulations each capped at 1 ns, already represent a significant milestone for MLPs^12,16^. However, we decided to challenge our best-performing models further by conducting 50 independent NVT simulations at 500 K and increasing the maximum simulation time to 10 ns.

Figure 4d, c report the cost (bottom row) and sampling (top two rows) achieved by these extended FFLUX simulations. Remarkably, each run completed the maximum simulation time of 10 ns, resulting in an aggregated simulation time of 0.5 microseconds (μs) or 2 billion simulation timesteps. Regarding the deployment cost, each MD timestep (or iteration) took 1.99 ± 0.14 ms, 4.03 ± 0.07 ms, 4.10 ± 0.16 ms, and 4.23 ± 0.11 ms for MAL, GLY, ASP, and SER, respectively, using as many CPU cores as the number of atoms in each system. For reference, full completion of the 40 million timesteps (for each run) required approximately 22.1, 44.7, 45.5 h, and 47.0 h, respectively. Despite using standard CPU hardware, these timings already compete with or even outperform existing state-of-the-art MLPs deployed on more sophisticated hardware. For instance, the NeQUIP model of ASP was reported to achieve an average FPS (frame per second) of 8.4 on NVIDIA Tesla V100-PCIe GPUs. This deployment speed is approximately 30 times slower than our FFLUX model for the same system^12^. Figure 4d suggests that the larger the system, the more beneficial it is to allocate more CPU cores. Close inspection of the unbiased NVT samples reveals a varying number of metastable states for MAL, GLY, ASP, and SER. For example, SER’s sampling density landscape indicates the presence of a highly populated metastable state in the beta sheet region of the Ramachandran plot (top left quadrant) and other lower-density metastable states located in the other three quadrants. In contrast, GLY is mainly found in eight different states, two in each quadrant, one of which is barely visible, at the far left. Finally, a comparison of Fig. 4c and Fig. 2b and Figure S3 confirms the similarity between the 1 ns and 10 ns samples.

### Beyond the robustness of atomic IQA energy models

After demonstrating the exceptional robustness of MF5-based atomic energy models, we assessed their ability to reproduce known stationary points using alanine dipeptide (AD) as a model system. For this purpose, we trained FFLUX models on 7000 AD geometries with IQA atomic energies computed at the B3LYP/def2-TZVP level. For each of the three lowest-energy AD gas-phase conformers, namely the C5, C7ax, and C7eq conformers, we generated 25 starting geometries (SGs) by applying random Cartesian displacements of up to 0.25 Å to an optimized structure of each conformer. Then, FFLUX geometry optimizations were performed within the atomic simulation environment (ASE)^27^ using an in-house FFLUX calculator and the BFGS (Broyden–Fletcher–Goldfarb–Shanno) quasi-Newton algorithm. For comparison, reference optimizations were carried out at the same level using the Berny algorithm as implemented in GAUSSIAN16^28^. All optimization runs were terminated when the maximum atomic force dropped to a value of 0.00045 Ha/Bohr (0.023 eV/Ǻ in ASE).

Figure S6 illustrates the structural (S6a) and energetic (S6b) diversity of the starting geometries (SGs). These high-energy structures span an RMSD range of 0.38 Å (0.29 Ǻ considering only heavy atoms). The most unstable configurations are located more than 1500 kcal mol^−1^ (6276 kJ mol^−1^) above the nearest minimum on the potential energy surface (PES), posing a substantial challenge for geometry optimization. Not surprisingly, GAUSSIAN16 could not optimize 3 of the 25 C7eq-like SGs, as the optimization did not proceed beyond the initial checks due to excessively short interatomic distances. It also failed to converge for one of C7ax-like SGs. These geometries were therefore excluded from subsequent analyses, although FFLUX still managed to relax them. According to Fig. S6d most of the optimization work occurs within the first 20 steps, during which the maximum atomic forces predicted by FFLUX models dropped from initial values as high as 1000 kcal mol^−1^ Ǻ^−1^ to roughly 1.0 kcal mol^−1^ Ǻ^−1^.

**Fig. 5:**
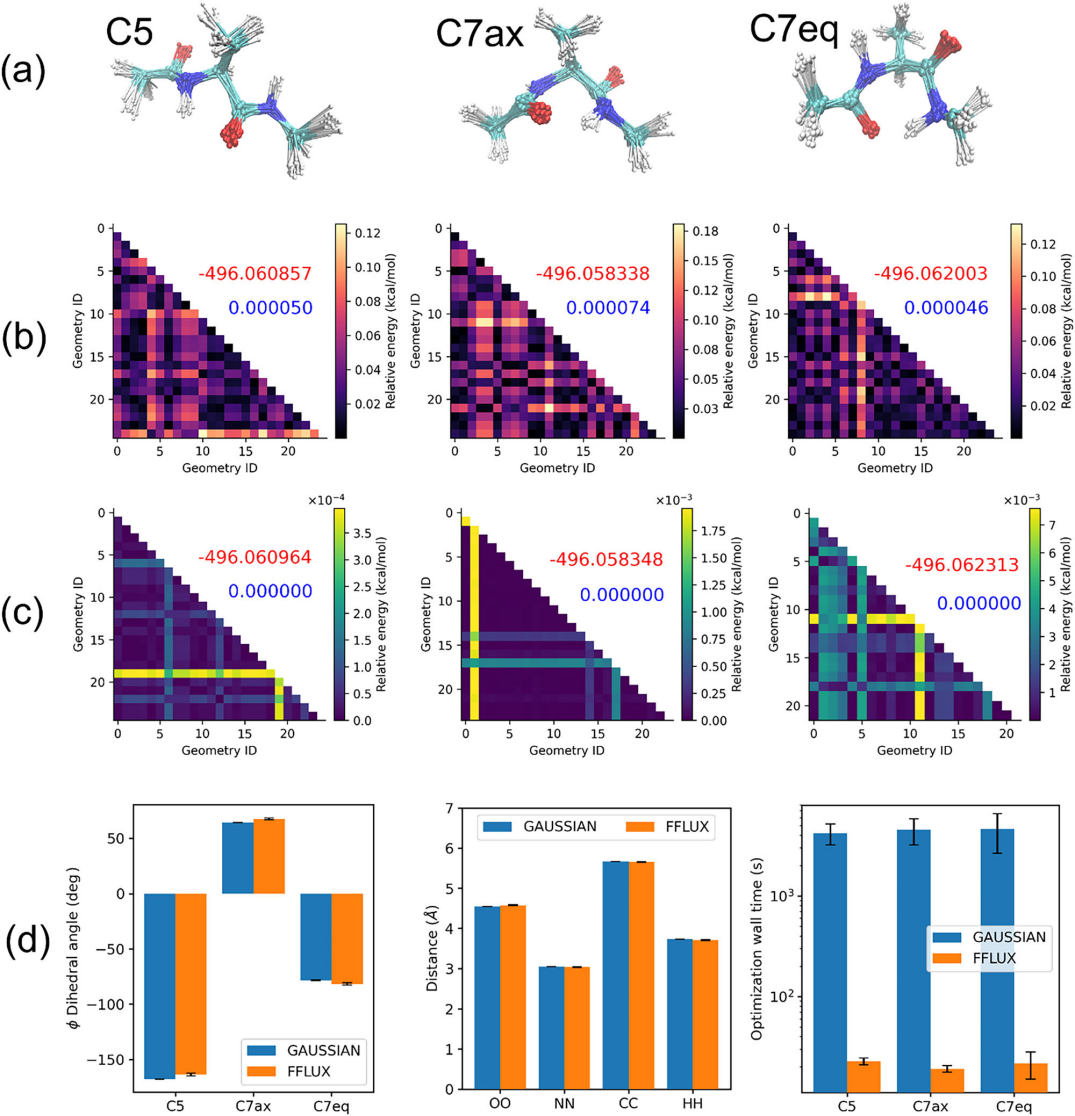
Optimization of alanine dipeptide structures using robust atomic energy models. **a** Starting geometries (SGs) for the optimization of alanine dipeptide (AD) structures. The cyan, white, red, and blue colors are used to indicate carbon, hydrogen, oxygen, and nitrogen atoms, respectively. **b**, **c** Energetic diversity of AD structures optimized by FFLUX and GAUSSIAN16, respectively. The numbers in red and blue on each subplot respectively indicate the average and the standard deviation of the potential energy. The first, second, and third columns correspond to the C5-like, C7ax-like, and C7eq-like structures. Prediction errors and relative energies between conformers were estimated based on the average values (in red). **d** Structural similarity between GAUSSIAN and FFLUX-optimized structures, as well as the optimization cost: (left) predicted dihedral angles of C5, C7ax, and C7eq conformers; (middle) predicted distances between pairs of selected atoms. The CC distance corresponds to the terminal C atoms, while HH indicates the NH…HN contact; (right) optimization cost (wall times) expressed in seconds. The *y* axis is log-scaled for visibility. We note that FFLUX atomic energy models (deployed in ASE) managed to optimize all 75 geometries, including the four for which GAUSSIAN16 could not proceed with the optimization or failed to converge for.

Figure 5 and Tables S2–S4 summarize the optimization results obtained with FFLUX and GAUSSIAN16. While both approaches consistently converge to the nearest local minimum, FFLUX models seemed more sensitive to the nature of the SG. This is reflected in the larger, but still negligible, variations in the potential energies (Fig. 5a, b) and geometries (Table S3) predicted by FFLUX in comparison to GAUSSIAN16. However, despite this sensitivity, FFLUX models reproduced the potential energies of the three conformers reasonably well, with the largest deviation estimated at 0.2 kcal mol^−1^ (0.8 kJ mol^−1^) for C7eq. They also correctly identified C7eq as the global minimum, positioned 0.7 kcal mol^−1^ (2.9 kJ mol^−1^) and 2.3 kcal mol^−1^ (9.6 kJ mol^−1^) below C5 and C7ax, respectively. Note that these predicted energy gaps are in very close agreement with the reference values of 0.8 kcal mol^−1^ (3.3 kJ mol^−1^) and 2.5 kcal mol^−1^ (10 kJ mol^−1^) for C5 and C7ax, respectively. From a structural perspective, FFLUX models managed to reproduce key internal coordinates of the three conformers with good accuracy, including $\phi$ and $\psi$ dihedral angles within 3° to 10°, and selected interatomic distances within 0.05 Å. On average, the RMSDs between FFLUX and GAUSSIAN-optimized structures were estimated at 0.09 Ǻ, 0.10 Ǻ, and 0.06 Ǻ for C5, C7ax, and C7eq-like structures, respectively (see Table S4), suggesting that atomic energy models can reliably reproduce known stationary points on a target potential energy surface (PES). Finally, FFLUX offers a two-order-of-magnitude reduction (~200x) in computational cost relative to GAUSSIAN16.

## Discussion

MLPs are usually benchmarked within their interpolation regime^29^, i.e., by testing them on geometries that look similar enough to those they were trained on. Unfortunately, with this approach, it is not easy to properly evaluate the effect of seemingly trivial control parameters that only affect the asymptotic behavior of a model. One such control parameter is the prior mean function *m* of a GP. Indeed, when the test and training data do not correlate well, such as in the extrapolation regime, the GP posterior mean (prediction) converges^30^ toward *m*. According to the literature, *m* is usually set to zero based on the assumption that all useful prior knowledge can be safely incorporated into the kernel simply by optimizing the kernel parameters^30,31^. However, when modelling physical phenomena, this widely adopted design choice can be dangerous to the point of causing unphysical predictions^32^. Although such unrealistic and certainly erroneous predictions can be alleviated or even prevented with carefully chosen and educated mean functions^33^, no study has hitherto investigated the impact of *m* on the robustness of GP-based MLPs.

The current study has not only contributed to filling a literature gap but also demonstrated that the mean function *m* of a GP-based MLP dictates its robustness. More specifically, we have proven that shifting *m* toward high-energy states yields very robust models. This finding implies that, given a decent training set and a molecular descriptor, how one chooses *m* will determine whether a GP-based simulation will crash instantly or run forever. In our attempt to rationalize the robustness of our GP-based atomic energy models, we found that they draw their robustness from the propensity to predict restoring forces that prevent the explosion or implosion of molecules. According to Stocker and coworkers, being able to prevent unphysical configurations in a simulation is a key characteristic of any robust MLP^26^. The fact that our MF5-based models fulfil this condition validates their unprecedented robustness at and beyond room temperature. To the best of our knowledge, this is the first time atomic IQA energy models have been capable of such robustness, achieving 0.5 microseconds of aggregated simulation time. Furthermore, unlike classical force fields, where robustness is sometimes achieved at the expense of reduced flexibility due to fixed topologies (predefined molecular connectivity with constraints on bond lengths), our models are robust without sacrificing flexibility.

We emphasize that, in addition to the choice of mean function, our atomic energy models also benefit from the fundamental quantum mechanical knowledge embedded in them. Such prior knowledge serves as an inductive bias, protecting the models against arbitrary variations of local energies in the extrapolation regime^34^. Furthermore, since topological atoms are the most transferable sub-systems that can be defined in real space^35^, FFLUX models inherit this physical transferability. The design of FFLUX around topological atoms sets it apart from existing Behler-Parrinello-like MLPs, which often suffer from the arbitrary variation of local energies^36^. This undesirable variation is a consequence of a key design choice common to all Behler-Parrinello-like MLPs, namely the fact that they allow the learning architecture to “dissect” the total energy of a system into site energies that have no physical meaning. Unlike the physics-informed QTAIM/IQA energies^37^, which are determined by quantum mechanical laws and with physical meaning naturally derived from the intra-atomic, Coulomb, and exchange-correlation components (see Eqs. 2 and 3 in the Methods section), the site energies of Behler-Parrinello-like MLPs can take on arbitrary values that appear to show little to no correlation with QTAIM/IQA energies^37^. Overall, the current approach paves the way for more challenging in-bulk simulations, where the robustness of our reported intramolecular models will be leveraged to counteract the deficiencies of sub-optimal intermolecular potentials.

In addition to their exceptional robustness, FFLUX atomic energy models can also reproduce known stationary points on a target PES (here illustrated through the optimization of AD). The fact that these models are not explicitly trained on atomic forces proves that the analytical forces obtained from the predicted IQA energies are sufficiently accurate to enable both stable molecular dynamics simulations and reliable geometry optimizations. While this work has demonstrated the unique performance of atomic energy models, we note that the FFLUX methodology^17,18^ is still under active development. Current research efforts focus on the explicit learning of electron-correlated atomic energies and on the design of an affordable, expressive, and transferable descriptor. The incorporation of electron correlation in atomic energy models is expected to be particularly important for condensed-matter applications, where correlated models will substitute empirical Lennard-Jones potentials and capture dispersion interactions. Given the high computational cost of IQA calculations, we have recently been exploring various ways to accelerate the generation of reference data for model training. Our preliminary investigations have revealed that delta-learning can be relied on to obtain CCSD(T)-quality topological electron-correlated data at substantially reduced cost, without any appreciable degradation in the accuracy of atomic correlation energies^38^. Finally, the ongoing work on the design of a local descriptor compatible with atomic IQA energies will enable transferability to systems beyond those included in the training set.

## Methods

### Interacting quantum atoms

The quantum theory of atoms in molecules (QTAIM)^39^ offers a means to partition any molecular system into space-filling atomic basins, Ω, following the gradient field of the electron density, ∇ρ. This mathematically rigorous process yields a set of physically transferable^35^ quantum topological atoms, each occupying a region in space bounded by a set of interatomic surfaces, each of which is defined by Eq. 1, where this equation is understood to be valid for *all* points on that surface,

1 $$ \nabla \rho \, \left(\bf r\right) \cdot \bf n\, \left(\bf r\right)\,\tt=0$$

where **n**(**r**) is a vector normal to the interatomic surface at position **r**.

Every molecular configuration is fully characterized by a unique set of atomic energies, denoted as $\left\{{E}^{\Omega }\right\}$. Each atomic energy can be estimated based on the atomic virial theorem^40^. However, this QTAIM energy decomposition approach only holds for stationary points on the PES. But then again, we proposed the use of six-dimensional volume integration, over two atoms simultaneously, in order to calculate the potential energy independently from the kinetic energy, thereby making the dependence on the virial theorem obsolete^41^. This advance inspired another research group to develop the exact energy partition we use in this article. Indeed, by exploiting the first- and second-order reduced density matrices, Blanco and coworkers showed twenty years ago that the total energy, E, of any system could be decomposed into atomic contributions^20^. Their formalism, known as the Interacting Quantum Atoms (IQA) approach, generates physics-informed atomic energies that capture all physical interactions within a system and recover the total energy of the system^42^. The total energy E of any system can be written as:

2 $$E={\sum }_{A}{E}_{{IQA}}^{A}={\sum }_{A}\left({E}_{{intra}}^{A}+\frac{1}{2}{\sum }_{B\ne A}{V}_{{inter}}^{{AB}}\right)$$

3 $${V}_{{inter}}^{{AB}}={V}_{{cl}}^{{AB}}+\,{V}_{{xc}}^{{AB}}$$

where the utmost left sum runs over all topological quantum atoms. The energy of each topological atom *A* is partitioned into an intra-atomic (self) energy ${E}_{{intra}}^{A}$ and an interaction energy ${V}_{{inter}}^{{AB}}=\frac{1}{2}{\sum }_{B\ne A}{V}_{{inter}}^{{AB}}$. According to Eq. 3, ${V}_{{inter}}^{{AB}}$ can be further decomposed into a Coulombic and an exchange-correlation component.

Due to numerical integration errors, Eq.2 is not exactly satisfied. The difference between the reconstructed molecular IQA energy and the DFT or wavefunction energy is known as the recovery error. If not properly accounted for, the missing part of the molecular energy will invariably introduce a systematic, albeit negligible, error in the calculation of atomic forces. To address this issue, in this work, we apply a correction to the raw atomic IQA energies, such that they fulfil Eq. 2. For a given geometry *G* and an atom *A*, the corrected atomic IQA energy is calculated by Eq. 4,

4 $${E}_{{IQA}}^{G,A,{corr}}={E}_{{IQA}}^{G,A,{raw}}+\Delta E\times \left[\frac{{E}_{{IQA}}^{G,A,{raw}}}{{E}_{{IQA}}^{G,{mol},{raw}}}+\left(\left|{L}_{A}\right|-\frac{{\sum }_{A}\left|{L}_{A}\right|}{{N}_{{atoms}}}\right)\right]$$

where $\Delta E$ is the recovery error, ${N}_{{atoms}}$ is the number of atoms in the system, and $\left|{L}_{A}\right|$ is the absolute value of the integration error of an atom *A* in geometry *G*. It is easy to prove that ${\sum }_{A}{E}_{{IQA}}^{G,A,{corr}}={E}^{G}$ where ${E}^{G}$ is the DFT or wavefunction energy of *G*.

### FFLUX atomic energy models

FFLUX’s Gaussian process regression (GPR) models are trained on atomic energies that correspond to Eqs. 2 and 4. The training of these models involves the optimization of several hyperparameters following a process we briefly outline in the next section. Assuming that all GPR models have already been trained, Eq. 5 can be used to predict the total electronic energy of an atom *A* in a given geometry *j* :

5 $${\hat{E}}_{{IQA}}^{A,j}={m}_{A}+{\sum }_{i=1}^{{N}_{{train}}}{\omega }_{i}{k}_{{ij}}\left({{{\boldsymbol{R}}}}^{{{\boldsymbol{i}}}},{{{\boldsymbol{R}}}}^{{{\boldsymbol{j}}}}\right)$$

where the hat above *E* stands for “predicted”. The sum in Eq. 5 runs over all the training geometries, ${\omega }_{i}$ is the i^th^ regression weight, and ${k}_{{ij}}$ is a kernel aimed at measuring the similarity between the test geometry *j* and the i^th^ geometry in the training set. The set of coordinates **R** is the system’s representation, which in this case, is a vector of input features computed using the atomic local frame (ALF) descriptor explained elsewhere^43^. Based on Eqs. 2 and 5, the total energy of a system can be obtained *via* reconstruction as shown in Eq. 6,

6 $${\hat{E}}_{{IQA}}^{j}=M+{\sum }_{A}{\sum }_{i=1}^{{N}_{{train}}}{\omega }_{i}{k}_{{ij}}\left({{{\boldsymbol{R}}}}^{{{\boldsymbol{i}}}},{{{\boldsymbol{R}}}}^{{{\boldsymbol{j}}}}\right)$$

where $M={\sum }_{A}{m}_{A}$. In this work, we consider five different definitions of ${m}_{A}$, shifted from low to high-energy states of individual topological atoms. This choice was motivated by the need to investigate the effect of the prior mean function on the asymptotic behavior of atomic energy GPR models. When shifting ${m}_{A}$ toward high-energy states, we made sure these mean functions remain reasonably physical, unlike the common zero mean function.

7 $${m}_{A}=\mu \left\{{E}_{{IQA}}^{A}\right\}$$

8 $$\,{m}_{A}=\mu \left\{{E}_{{IQA}}^{A}\right\}+1.0\sigma \left\{{E}_{{IQA}}^{A}\right\}$$

9 $$\,{m}_{A}=\max \left\{{E}_{{IQA}}^{A}\right\}$$

10 $${m}_{A}=\max \left\{{E}_{{IQA}}^{A}\right\}+\,1.0H\left\{{E}_{{IQA}}^{A}\right\}$$

11 $${m}_{A}=\max \left\{{E}_{{IQA}}^{A}\right\}+\,5.0H\left\{{E}_{{IQA}}^{A}\right\}$$

where *μ*,*σ*, max and *H* are the mean, the standard deviation, the maximum value and the range of reference atomic IQA energies, respectively. The acronym MFn ($n\in \left\{{\mathrm{1,2,3,4,5}}\right\}$) is chosen to refer to the five mean function types defined in Eqs. 7 to 11, respectively, so MF5 corresponds to Eq. 11.

### Metaheuristic hyperparameter optimization

The training of each FFLUX atomic energy model involves the optimization of 3${N}_{{atoms}}$-4 hyperparameters, with ${N}_{{atoms}}$ being the number of atoms in the system. Among these hyperparameters, 3${N}_{{atoms}}$ − 6 are kernel parameters denoted as $\left\{{\theta }_{k}\right\}$. The remaining hyperparameters are the regularization noise ${\sigma }_{n}^{2}$ and the kernel pre-factor, ${\sigma }_{f}$. Unlike the pre-factor, which is estimated based on the user’s instructions (details see program documentation) and the dataset at hand, both $\left\{{\theta }_{k}\right\}$ and ${\sigma }_{n}^{2}$ are iteratively refined using an enhanced grey wolf optimizer (GWO)^44^ implemented in our in-house GPR engine, FEREBUS^45,46^.

In the GWO search mechanism, a certain number of working agents scrutinize the hyperparameter space (HS) guided by well-defined communication rules^47^. Every GWO agent ${{{\boldsymbol{\theta }}}}^{{{\boldsymbol{j}}}}$ is encoded as a vector of length 3${N}_{{atoms}}$- 4 and constitutes a candidate solution. Solutions are ranked based on their proximity with respect to an unknown optimal solution that minimizes the loss function in Eq. 12,

12 $${{{\mathcal{L}}}}_{A}^{2}=\frac{1}{{N}_{{val}}}{\sum }_{v=1}^{{N}_{{val}}}{\left[{E}_{{IQA}}^{A,v}-\,{\hat{E}}_{{IQA}}^{A,v}\left({{{\boldsymbol{\theta }}}}^{{{\boldsymbol{j}}}}\right)\right]}^{2}$$

where ${N}_{{val}}$ is the number of validation geometries, ${E}_{{IQA}}^{A,v}$ and ${\hat{E}}_{{IQA}}^{A,v}$ are respectively the reference and predicted energies of an atom *A* in the V^th^ validation geometry. The three best solutions are called leader agents and denoted by α, β and δ. Their positions serve to guide the collective movement of the non-leader GWO agents (ω) within the HS. The position of a given ω agent ${{{\boldsymbol{\theta }}}}^{{{\boldsymbol{\omega }}}}$ is updated using Eq. 13,

13 $${\theta }_{d}^{\omega }\left(t+1\right)=\frac{1}{3}{\sum }_{l}\left[{\theta }_{d}^{l}\left(t\right)-{A}_{l,d}\left(t\right){D}_{d}^{l,\omega }(t)\right]$$

14 $${D}_{d}^{l,\omega }(t)=\left|{C}_{l,d}(t){\theta }_{d}^{l}\left(t\right)-{\theta }_{d}^{\omega }(t)\right|$$

where *t* is the current iteration, $l\in \left\{\alpha ,\beta ,\delta \right\}$, and *d* is the dth dimension of the HS. The agent-specific perturbation matrices *A* and *C* are respectively defined in Eqs. 15 and 16, where the ${r}_{1}$ and ${r}_{2}$ random numbers are uniformly sampled between 0 and 1. The control parameter $a\left(t\right)$ is decreased linearly from 2 to 0 along the optimization path.

15 $${A}_{l,d}\left(t\right)=2a\left(t\right){r}_{1}-a(t)$$

16 $${C}_{l,d}\left(t\right)=2a\left(t\right){r}_{2}$$

To accelerate the convergence, our GWO implementation^44^ incorporates a new operator $\mho$ in the search mechanism of vanilla GWO^47^. This operator promotes a user-specified number of lucky non-leader agents $\left\{{\omega }^{* }\right\}$ towards the centroid of the leaders’ positions L.

17 $${{\rm{\mho }}}\left[{\theta }^{{\omega }^{* }}\left(t+1\right)\right]=\left(1+\varepsilon \right)\times L\left(t+1\right)$$

In Eq. 17, $\varepsilon$ is a small random number between 0 and 0.25. Finally, an elitism check is performed every iteration to make sure the agents only move toward better solutions.

### Prediction of atomic forces

Since FFLUX models are not currently trained on atomic forces, the latter must be computed on the fly using carefully derived analytical formulas^48^. Accordingly, the force acting on an atomic *A* along a global axis $\kappa$ is computed using Eq. 18,

18 $${\hat{F}}_{A,\kappa }^{j}=-\frac{\partial {E}^{j}}{\partial {\kappa }_{A}}=-\frac{\partial {E}^{j}}{\partial {{{\boldsymbol{R}}}}^{{{\boldsymbol{j}}}}}\frac{\partial {{{\boldsymbol{R}}}}^{{{\boldsymbol{j}}}}}{\partial {\kappa }_{A}}$$

where $\kappa \in \left\{x,y,z\right\}$ and ${E}^{j}$ is defined in Eq. 6. The $\frac{\partial {{{\boldsymbol{R}}}}^{{{\boldsymbol{j}}}}}{\partial {\kappa }_{A}}$ terms (stored in the so-called *B* matrix) ensure the conversion from the local frame in which the models are trained to the global frame, while the $\frac{\partial {E}^{j}}{\partial {{{\boldsymbol{R}}}}^{{{\boldsymbol{j}}}}}$ terms are defined as:

19 $$\frac{\partial {E}^{j}}{\partial {{{\boldsymbol{R}}}}^{{{\boldsymbol{j}}}}}=\,{\sum }_{A}{{{\boldsymbol{\omega }}}}^{A}\left(\frac{\partial {{\boldsymbol{k}}}}{\partial {{{\boldsymbol{R}}}}^{{{\boldsymbol{j}}}}}\right)={\sum }_{A}{\sum }_{i=1}^{{N}_{{train}}}{\omega }_{i}^{A}\left(\frac{\partial {k}_{{ij}}\left({{{\boldsymbol{R}}}}^{{{\boldsymbol{i}}}},{{{\boldsymbol{R}}}}^{{{\boldsymbol{j}}}}\right)}{\partial {{{\boldsymbol{R}}}}^{{{\boldsymbol{j}}}}}\right)$$

where the right-most derivative depends on the choice of covariance function or kernel. In this work, we make use of the composite kernel defined in Eq. 20,

20 $${k}_{{ij}}\left({{{\boldsymbol{R}}}}^{{{\boldsymbol{i}}}},{{{\boldsymbol{R}}}}^{{{\boldsymbol{j}}}}\right)={\sigma }_{f}\times \exp \left(-{\sum }_{d=1}^{{N}_{{feats}}}{\theta }_{d}{\Phi }_{d}\left(i,j\right)\right)$$

In Eq. 20, ${\theta }_{d}$ is the d^th^ kernel parameter. The piecewise function ${\Phi }_{d}\left(i,j\right)$ considers the nature of ALF features and is calculated according to Eq. 21,

21 $${\Phi }_{d}\left(i,j\right)=\left\{\begin{array}{c}{\sin }^{2}\left[0.5\times \left({R}_{d}^{i}-{R}_{d}^{j}\right)\right]\,{if}\,d > 3\,{and}\,{MOD}\left(d,3\right)=0\\ {\left({R}_{d}^{i}-{R}_{d}^{j}\right)}^{2}\,{otherwise}\hfill\end{array}\right.$$

where MOD is the modulo operator.

### Sampling

The PES of each system of interest was sampled by performing 300 K WTMetaD simulations at the GFN2-xTB^24^ level in the canonical NVT ensemble. Each simulation was propagated for 1 ns with a timestep of 0.5 fs, and the geometries were sampled every 100 fs (200 fs for MAL and ASP). The simulation temperature was maintained at around 300 K, using the Langevin thermostat with a friction coefficient of 0.01 fs^−1^. The Langevin thermostat was chosen to promote efficient sampling by injecting controlled randomness into the dynamics. Note that stochastic thermostats such as the Langevin thermostat promote efficient sampling by *injecting controlled randomness into the dynamics*. This helps the system overcome energy barriers, escape metastable states, and explore the phase space more thoroughly. Therefore, they naturally seem more appropriate when it comes to building training sets. By using the Langevin thermostat in the sampling phase, we aimed at improving the coverage of conformational space through the introduction of random noise. The success of this choice is reflected in the decent sampling achieved (Fig. S1).

For GLY and SER, the $\phi$ and $\psi$ torsional angles around the central asymmetric carbon were chosen as collective variables, and the bias potential was computed using a PLUMED^49^ interface with the Atomic Simulation Environment (ASE)^27^. The resulting configuration databases were sorted in terms of structural diversity using our diversity-aware sub-sampler (DAS). The latter is a farthest-point selection procedure that iteratively selects a subset of geometries that best preserves the structural diversity of an existing population based on the Hausdorff root-mean-square deviation between the sample pool and the set of selected geometries. The DAS procedure is initialized from a seed geometry, which we choose here to coincide with the optimized structure of the system of interest. More details, including the pseudocode of the DAS procedure, are provided in the last section of the S1. To generate the target atomic IQA energies, topological calculations were performed at the B3LYP/6-31 + G(d,p) level for GLY and SER, and B3LYP/6-31 + + G(d,p) level for MAL and ASP using the AIMAll19 program^50^.

### Model training

All the models were trained using the most recent version of our in-house GPR engine, FEREBUS^44,46^. For each system, we trained 10 different sets of atomic energy models. These corresponded to the five mean types and two training set sizes, namely 1000 and 5000 geometries. An internal validation set of 1000 geometries was selected to direct the hyperparameter optimization, which was propagated for 200 iterations using small teams of 50 working agents. The hyperparameter space was defined as [0, 1] and [10^−10^, 10^−4^] for the $\left\{{\theta }_{k}\right\}$ and ${{\sigma }_{n}}^{2}$ hyperparameters, respectively, while the kernel pre-factor was fixed throughout the search and its value was calculated as ${\sigma }_{f}={\left({N}_{{train}}\times {N}_{{feats}}\right)}^{0.5},$ where ${N}_{{train}}$ and ${N}_{{feats}}$ are the number of training points and input features, respectively. A random update of the hierarchy ladder^44^ was performed every 5 iterations by choosing 5 ${\omega }^{* }$ wolves and shifting their positions toward the centroid of the three leaders. This design choice aims to accelerate the convergence of candidate solutions, without turning the process into a random search.

### Model validation and deployment

Once trained, each model was initially tested on an unseen set of 1000 geometries. As customary, mean absolute errors (MAEs) were calculated as a preliminary measure of the models’ performance. Because these models were trained on atomic energies, molecular MAEs were calculated through reconstruction. The previous static tests were followed by the deployment of the trained models in NVT simulations using the DL-FFLUX program^17^. The latter in-house program is written as an extension of the DL-POLY4 simulator^51^. For each system of interest, we considered 10 different SGs and carried out, for each SG, four simulations, one at 300 K, 500 K, 800 K, and 1000 K. The simulation temperature was maintained around the chosen value using a Nosé-Hoover thermostat. This deterministic thermostat was chosen to ensure that the correct canonical distribution is sampled while minimally perturbing dynamical correlations. Unlike stochastic thermostats (i.e., Langevin), deterministic thermostats propagate the system’s dynamics without introducing random noise. Consequently, they generate the correct canonical distribution while minimally perturbing dynamical correlations, making them ideal for production runs. This is why the Nosé-Hoover thermostat was used for production runs.

With a timestep of 0.25 fs, each run was propagated for 4 million timesteps to achieve 1 ns of simulation time. A given simulation was considered to be unstable if any bond in the molecule was stretched or compressed by more than 1.65 times its equilibrium length. In other words,

22 $$S\left(T,{{\mathcal{M}}}\right)=\tau \,{{\rm{if}}}\,\forall b\epsilon B,\,\exists {b}_{i}\,{{\rm{such\; that}}}\,{b}_{i} > 1.65{b}_{i}^{0}\,{{\rm{or}}}\,{b}_{i} < {b}_{i}^{0}/1.65$$

where $S\left(T,{{\mathcal{M}}}\right)$ is the stability of a simulation actioned by a model *M* at the temperature *T*, τ is the simulation time, G is the τ^th^ simulation snapshot, $B=\left\{{b|b}\in G\right\}$ is the set of bonds, ${b}_{i}$ is the bond of interest, and ${b}_{i}^{0}$ is the corresponding equilibrium bond length as in the optimized structure of the system. The first and second inequalities in the stability condition shown in Eq. 22 distinguish explosive from implosive crashes.

Having evaluated the stability of each run, the robustness of the underlying model was computed using Eq. 23:

23 $$R\left(T,{{\mathcal{M}}}\right)=\frac{1}{{N}_{{sim}}}{\sum }_{i=1}^{{N}_{{sim}}}{S}_{i}\left(T,{{\mathscr{M}}}\right)$$

where ${S}_{i}\left(T,{{\mathcal{M}}}\right)$ is the stability of the *i*th simulation and ${N}_{{sim}}$ is the number of equivalent simulations (in our case, ${N}_{{sim}}$ = 10).

## Supplementary information

Supplementary Information

## Data Availability

Most of the data supporting this work are either reported in the main or the supporting information, or stored in the following Mendeley data repository (10.17632/b75bjps2vw.1). These data include: (a) all the training and validation sets; (b) the atomic energy models; (c) starting geometries for the 1 ns and 10 ns FFLUX simulations; (d) unbiased MD samples from extended FFLUX simulations (>100,000 geometries for each molecule); (e) three videos showing the predicted atomic forces during the first picosecond of NVT simulations of MAL, ASP, and SER at 500 K and initiated from high-energy structures. The alanine dipeptide (AD) data, including the models, experiment output files, submission and post-processing scripts, are stored at https://github.com/popelier-group/AD_data.

## Code availability

The FEREBUS and DL_FFLUX programs are accessible at https://github.com/popelier-group/FEREBUS_CPU/ and https://github.com/popelier-group/DL_FFLUX-v3, respectively. A stable version of the FFLUX calculator for the atomic simulation environment can be found here https://github.com/popelier-group/FFLUX_ASE. The DL_FFLUX code can also be cited using the following 10.5281/zenodo.18713944.
