# Supplementary material for: Unprecedented robustness of physics-informed atomic energy models at and beyond room temperature
Source: Commun Chem. 2026 Mar 31;9:138. doi: 10.1038/s42004-026-01965-0 (PMC13039801; doi:10.1038/s42004-026-01965-0)
Supplement: Supplementary file 1 — Supplementary Information [file 42004_2026_1965_MOESM1_ESM.pdf]

SUPPORTING INFORMATION

Unprecedented robustness of physics-informed atomic energy models at and beyond room temperature

Bienfait Kabuyaya Isamura, Olivia Aten, Mohamadhosein Nosratjoo, and Paul Lode Albert Popelier

Department of Chemistry, The University of Manchester, Manchester, M13 9PL, United Kingdom

Email: paul.popelier@manchester.ac.uk

1. Datasets

In this section, we briefly discuss the quality of the datasets used for training the models. We first focus on the target atomic energies before discussing the structural diversity of our datasets.

1.1 Atomic IQA energies

As mentioned above, our atomic energy models were trained on topological IQA energies obtained through a numerically intensive and mathematically rigorous process. To assess the quality of the reference energies, we first examined the energetic deviation (ΔE) between the total IQA energies and DFT energies (also known as recovery error). We also inspected how the total charge of the system was reconstructed from QTAIM atomic charges by calculating the recovery charge Δq00. The results obtained are collected in Table **S1**.

Table S1: Quality of our QTAIM/IQA datasets. The recovery error thresholds are expressed in kcal/mol and milli-electron (me) for ΔE and Δq00, respectively. The column #ZSF specifies the number of geometries that we retained by the Z-score filter. For the filtered datasets, we provide in the bottom part of the Table the medians of the integration error distributions and separate the statistics into hydrogen and "heavy" (non-hydrogen) atoms. For each atom A, $\{L_A\}$ is the set of atomic integration errors, which contains both positive $\{L_A{}^{+}\}$ and negative $\{L_A{}^{-}\}$ values. We define the parameter $\zeta = \#\{L_A{}^{+}\}/\#\{L_A\}$, which indicates the ratio between the number of positive integration errors $\#\{L_A{}^{+}\}$ and the total number $\#\{L_A\}$ of computed integration errors for a given atom A.

System	**#Geom.**	**#(ΔE>1)**	**#(Δq00>1)**	**#(ZSF)**	**Removed**
GLY	10,000	3	234	6	232
SER	10,000	4	454	29	482
MAL	5,000	0	2	10	12
ASP	5,000	9	133	14	144
		GLY	SER	MAL	ASP
# Filtered geometries		9768	9518	4988	4856
$\text{median}^{\text{All}}$		3.1e-5	3.4e-5	2.4e-5	3.4e-5
median^{H}		2.4e-5	2.5e-5	2.0e-5	2.0e-5
$\text{median}^{\text{Heavy}}$		8.6e-5	8.4e-5	5.2e-5	7.6e-5
ζ^{all}		0.777	0.753	0.809	0.717
ζ^{H}		0.970	0.943	0.996	0.983
ζ^{Heavy}		0.562	0.546	0.661	0.553

It turns out that recovery energy errors lie within 1 kcal/mol for more than 99.8% of the initial geometries. Since every geometry with a recovery energy error ΔE > 1 kcal/mol also has a charge reconstruction error Δq_{00} > 0.001e, and many additional geometries exceed this Δq_{00} threshold without large ΔE errors, Δq_{00} serves as a more stringent filter for atomic IQA datasets. Indeed, the removal of outliers has been found to improve the convergence of various optimisers and enhance the overall performance of machine learning models[1]. Keeping in mind that not every noisy data point is an outlier, we herein call “outlier” any IQA energy value that is inconsistent with the other values[2].

Two separate but complementary filters were applied to remove possible outliers before training the models. The first filter consisted of removing all geometries in which the IQA energy of at least one of the atoms violated an absolute Z-score > 3 (see ZSF column in Table **S1**). Since outliers inflate both the mean and standard deviation of any distribution, the distributions of atomic IQA energies in this case, it was expected that the Z-score filter would struggle to detect some of them[2]. We thus required a second, more rigorous filter, capable of identifying any outliers missed by the first one. This consisted of filtering out all geometries with Δq00 greater than 1 me. As expected, the number of possible outliers increases with the system’s size, rising from 0.2 % to 4.8 % between MAL and SER. This trend is an indication of an increasing challenge in performing atomic integrations for larger systems, which can be attributed to the increasing number of confined atoms whose cusped boundaries complicate the radial integration[3].

Although the recovery energy ΔE was not explicitly incorporated in the filtering pipeline, all the geometries with the largest recovery errors were caught by the composite Δq000/ZSF filter and subsequently removed. These extremely unusual and tricky geometries also exhibited the largest atomic integration errors $max\{L_A\}$. However, we could not establish a strong correlation between ΔE and $max\{L_A\}$. This finding corroborates the results by Aicken and Popelier[4], who came to the same conclusion twenty-five years ago. The same authors also found that increasing the number of integration grids does not always lead to lower integration errors. As expected, hydrogen atoms exhibit the smallest atomic integration errors in the filtered geometries. Furthermore, the fact that the medians of $\{L_A\}$ values over the entire molecule remain lower than 1.0e-4 E_h is an indication of the high quality of our target IQA energies[5].

We aim to highlight an unexpected pattern regarding the sign of atomic integration errors. According to the ζ_H and ζ_{Heavy} parameters in Table **S1**, the integration errors of hydrogen atoms are mostly positive (ζ_H between 0.94 and 1.00), while a more even distribution in observed for “heavy” (i.e. non-hydrogen) atoms (ζ_{Heavy} between 0.55 and 0.66). The fact that this applies to both GLY and SER (obtained at B3LYP/6-31+G(d,p) level), as well as to MAL and ASP (obtained at B3LYP/6-31++G(d,p) level), suggests that this is a general pattern independent of the choice of basis set. Finally, although the recovery energy errors ΔE were negligible, it was necessary to correct the raw energies to prevent any systematic error in the prediction of atomic forces. To achieve this, we designed the empirical correction formula in Eq.**4** of the main text.

1.2 Structural diversity

We will now discuss the structural diversity of our datasets. We will first inspect the sampling achieved by the WTMetaD sampler (Figure **S1**), before comparing it with alternative standard MD samplers (Figure **S2**). Our analysis is based on the distributions of distances between selected pairs of atoms. Figure S1 demonstrates the enhanced sampling capability of WTMetaD. Compared to Figure **4** in the main text, the WTMetaD sampler (at 300 K) ensures an almost uniform coverage of ASP and MAL's conformational space, sampling high-energy geometries that could not be reached by the unbiased FFLUX sampler even at temperatures as high as 1000 K. The same is true for SER and GLY (better sampling *via* WTMetaD). This is not surprising: it just proves that increasing the simulation temperature of unbiased simulations is not as efficient as biasing the simulation's potential when it comes to sampling the conformational space of flexible molecules.

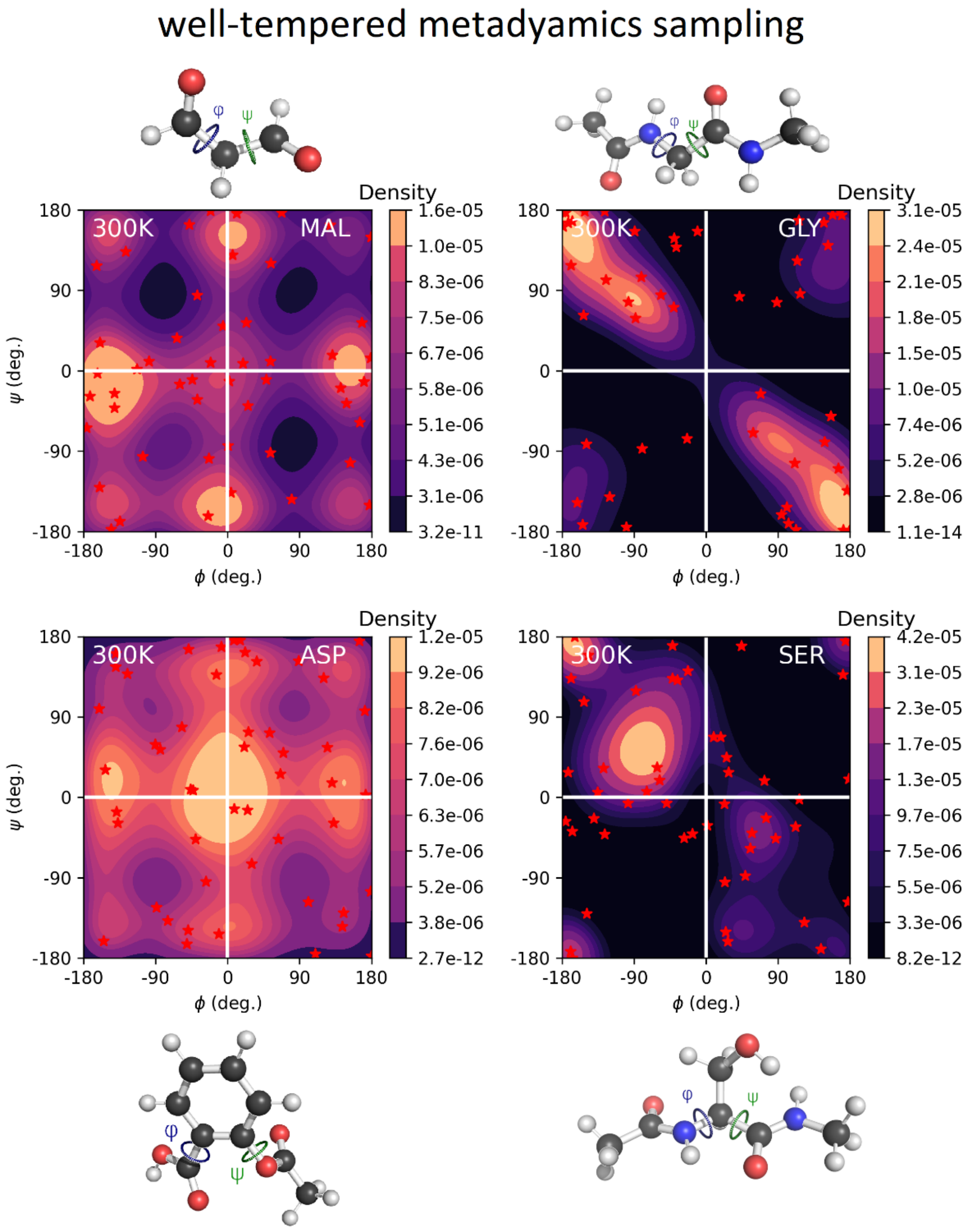

Figure S1. Well-tempered metadynamics samples of glycine (GLY), serine (SERINE), malondialdehyde (MAL) and aspirin (ASP) at 300 K. The GLY and SER samples contain 10,000 geometries each, while the MAL and ASP ones have 5,000 geometries each. The color bar indicates the sampling density. The ϕ and ψ torsional angles were used as collective variables in the biased MD simulations. These same angles are used as the coordinate axes in this figure to project and visualise the sampled configurations. The first 50 most diverse geometries, sorted using the DAS algorithm explained in section 5, are shown as red stars.

Figure **S2** shows that the C...C distance between terminal C atoms in the GLY and SER datasets varies over a range of 3 Å, namely between 4.5 Å and 7.5 Å. The corresponding distributions carry two bands located around $r_{C...C} \approx 5.5$ Å and $r_{C...C} \approx 7.0$ Å. These bands reflect the presence of both compact and extended states, respectively. Note also the reasonable density (≈ 0.2) between the left and right bands, which indicates a decent sampling of configurations along the transition path between the compact and extended metastable states.

For MAL we focus on the distance between the two O atoms, which varies continuously with the independent rotation of the two equivalent C-C-C-O dihedral angles. According to Figure **S2**, the O...O distance in the MAL dataset varies between 2.5 Å and 5.0 Å, and the associated distribution is maximised around $r_{O...O} \approx 4.0$ Å, a region occupied by W-*trans*-like structures. Also well-represented in the MAL dataset are the S-*trans* and *cis*-like structures that form the barely visible shoulders around $r_{O...O} \approx 4.5$ Å and $r_{O...O} \approx 3.0$ Å, respectively. Furthermore, the sampling density remains higher than 0.20 between 3.0 Å and 4.7Å, indicating the satisfactory coverage of the transition regions between the S-*trans* and *cis* regions via the W-*trans* region. We observe a lower representation of *cis*-like structures, which is consistent with the shorter distance and the interaction between the two O atoms[6, 7] considered as more repulsive according to the quoted literature.

The broader shape of the H...O curve of ASP is consistent with the full rotation of the carboxylic and acetoxy groups (see mist plots in Figure **S2**). The models reported in this study were trained on subsets of the full databases. These subsets were obtained by sorting the geometries in each database in terms of structural diversity, then selecting the first N_{train} geometries.

According to Figure **S2**, the subset curves present qualitative patterns that align almost perfectly with those of the full database. This observation confirms the aptitude of farthest-point sampling (FPS) algorithms[8], in this case, our diversity-aware sub-sampler (DAS), to identify representative subsets from an existing large collection of geometries. The DAS procedure selects common structures based on the Hausdorff root-mean-square deviation H(C,S) between the set of candidate geometries *C* and the temporary set of selected structures *S*. This process requires a seed point[8], which we chose to coincide with the optimised geometry of the molecule, thus making the whole process deterministic. Note that once the seed point (geometry) is chosen, the process always lead to the same set of most diverse geometries. This is not the case for a typical FPS algorithm in computer vision, where the seed pool (possibly more than one point) is randomly selected from the dataset, which makes the whole process not deterministic.

A final comment pertains to the comparison between standard MD samplers, more specifically, the GAFF[9] and GFN2-xTB[10] samplers on one hand, and the WTMetaD sampler on the other hand. As anticipated, the biased WTMetaD sampler achieves the most diverse and representative sampling of the target PES, while the semi-empirical GFN2-Xtb sampler outperforms the classical GAFF. Furthermore, increasing the simulation temperature appears more beneficial for GFN2-xTB, whose 500 K samples are already comparable to those of WTMetaD for both GLY and SER. In contrast, the GAFF sampler produces the least flexible dynamics, possibly because of the rigid and pre-defined form of the energy terms. The case of MAL stands out as the GAFF propagator is unable to escape the energetic well around the S-*trans* conformer (starting geometry). Finally, compact GLY and extended SER structures remain poorly represented in the 500 K GAFF ensemble, which also shows a negligible population of structures along the transition path between these states.

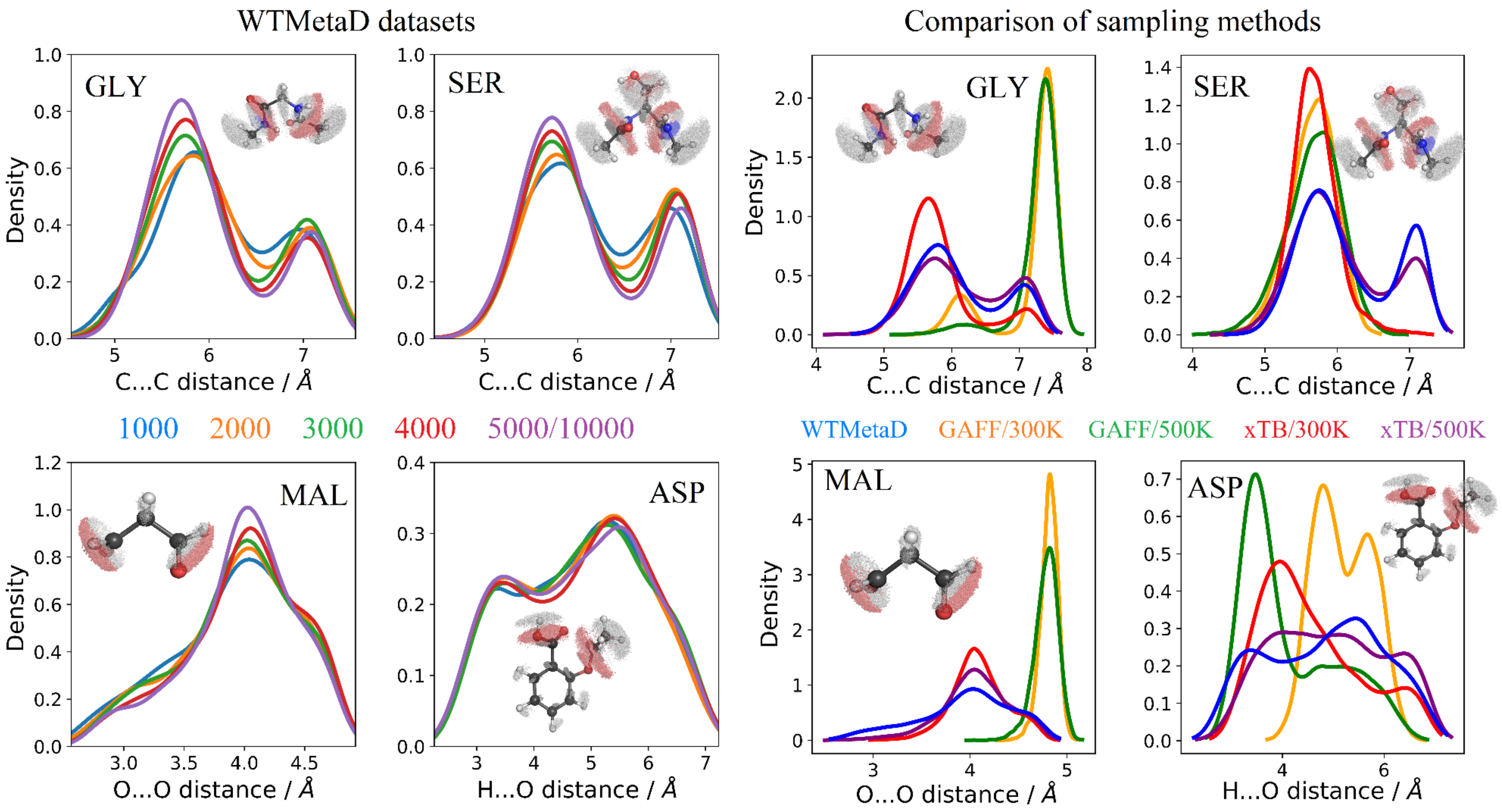

Figure S2. Distribution of selected pairwise distances in the WTMetaD and alternative datasets (GAFF and xTB) of the four systems of interest. WTMetaD datasets: the two carbon atoms in the glycine (GLY) and serine (SER) plots correspond to the terminal C atoms. For malondialdehyde (MAL) we considered the two O atoms, while for aspirin (ASP) we looked at the distance between the carboxylic H atom and the carbonyl O atom of the acetoxy group. All distances are expressed in Å and the curves are obtained via kernel density estimation using the Silverman method for estimating the bandwidth. The blue, orange, green and red curves respectively denote subsets of 1000, 2000, 3000 and 4000 geometries. The purple curve corresponds to the full database, i.e., 5000 for MAL and ASP, and 10,000 geometries for GLY and SER. The mist plots for WTMetaD datasets are also shown in the small ball-and-stick image in each of the eight panels.

2. NVT sampling of GLY and ASP using 1000-point GP-based atomic energy models deployed in DL_FFLUX

The GLY-1000-MF5 and ASP-1000-MF5 models were deployed in NVT simulations at 300 K, 500 K, 800 K and 1000 K. For each model and temperature, ten independent simulations were performed using structurally diverse starting geometries. The resulting trajectories were combined, then sub-sampled to obtain the sampling density plots shown in Figure **S3**. Notice that GLY is more flexible than SER (Figure **2b** for comparison). As anticipated, increasing the simulation temperature broadens the coverage of the configuration space, which is particularly true for ASP.

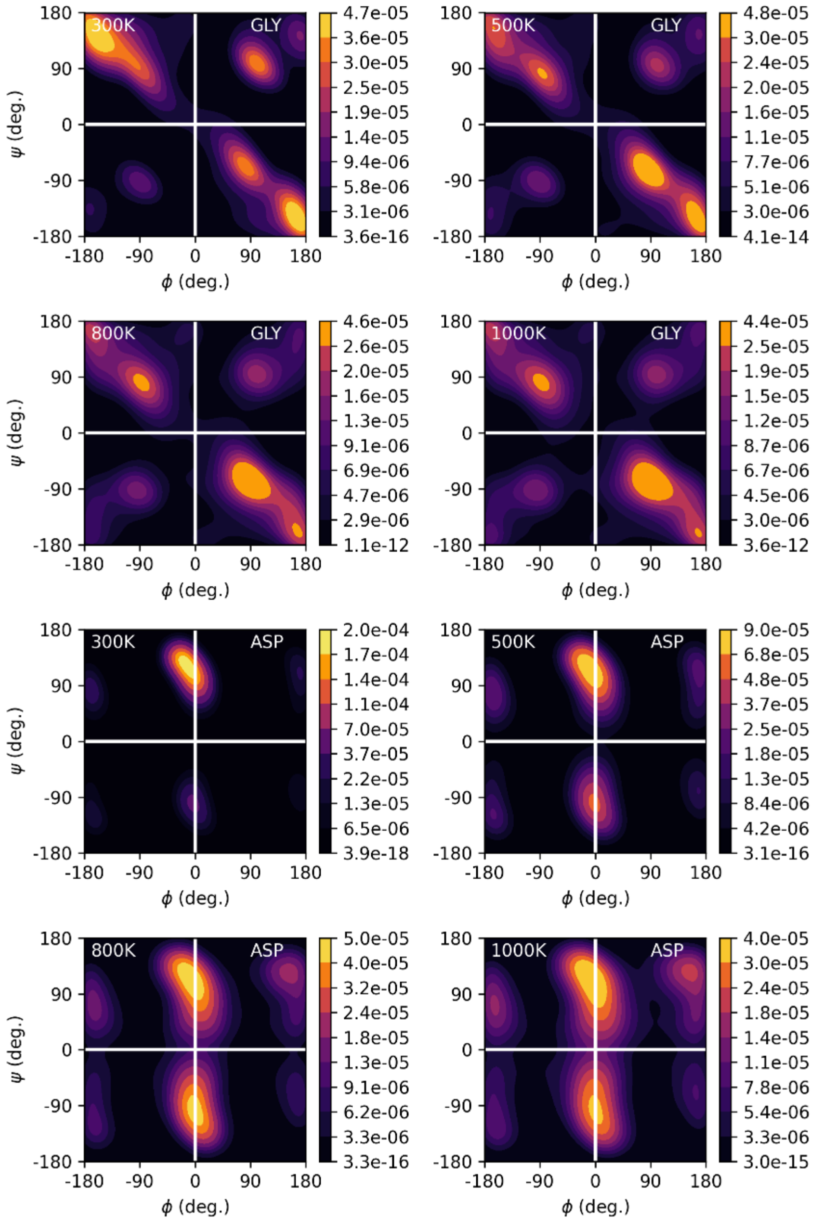

Figure S3. FFLUX molecular dynamics sampling of GLY (top 4 panels) and ASP (bottom 4 panels) configuration space at 300 K, 500 K, 800 K and 1000 K (using MF5-based models). The configuration samples are projected along the same directions as shown in Figure **4** in the main text. Each subplot was generated from a sample of 100,000 geometries, obtained by extracting 10,000 frames from each run at constant intervals of 400 timesteps.

3. Relaxation of high-energy aspirin geometries with unrealistic angles, dihedrals and bonds

In this section we present the results for two experiments (challenges 4 and 5) aimed at assessing the ability of the ASP-1000-MF5 model to relax high-energy geometries. The datasets for other challenges (1, 2 and 3), obtained by stretching and/or compressing different bonds in the optimised geometry of ASP, are provided as external resources at the following link (DOI: 10.17632/b75bjps2vw.1). The ASP-1000-MF5 model was deployed in an NVT simulation at 500 K using two unstable starting geometries (SGs). The first SG was generated by compressing four bonds from their equilibrium lengths to 0.9 Å. The second SG was obtained by manually perturbing two angles and two dihedral angles in the benzene ring. We chose two opposite C-C-C angles and set them to 145°, while the benzenic hydrogen atoms were taken out of the ring's plane by roughly 30°.

The resulting SGs are shown in Figure **S4** and lie 193.4 kcal/mol and 114.6 kcal/mol above the nearest local minima on the B3LYP/6-31++G(d,p) potential energy surface. Needless to say, these SGs are outside the training space of their respective models, and dealing with them constitutes a challenging test for these models. The results for the first SG are presented in the main text. Figure **S4** shows that the ASP-1000-MF5 model managed to amend the very short bonds and unrealistic angles and dihedrals in the same way it did for very long bonds. This substantial correction was achieved within 1 ps, and the simulation continued without crashing for another 1 ns. For SG2, the two perturbed C-C-C angles in the ring went from 145° to oscillating around 120°. These findings confirm the outstanding robustness and relaxation aptitude of the ASP-1000-MF5 model, which can deal with both stretched and compressed bonds, as well as unrealistic angles and dihedrals.

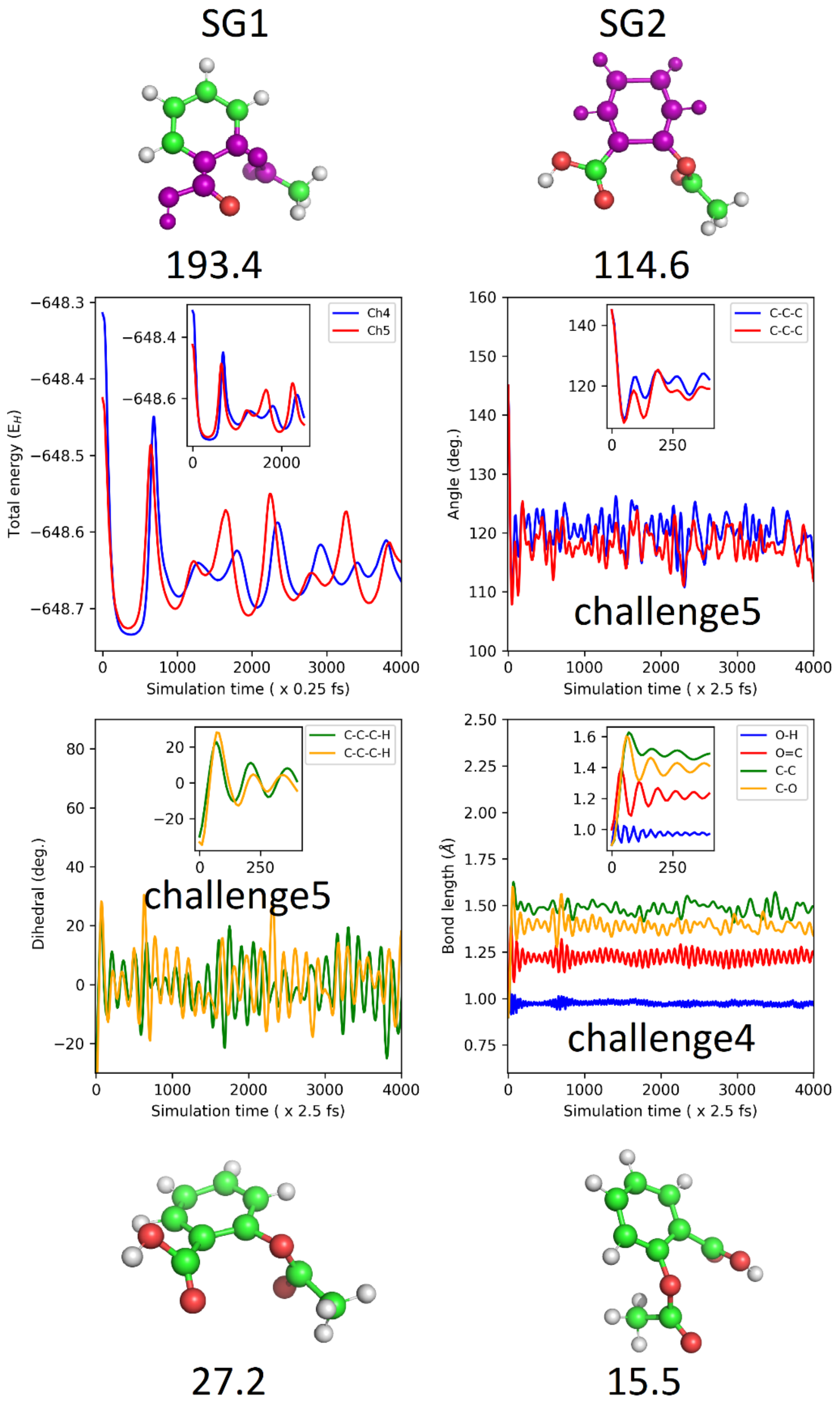

Figure S4. First 1 ps (for total energy) and first 10 ps for internal coordinates of 500 K NVT simulations of aspirin using “challenge4” (Ch4, SG1) and “challenge5” (Ch5, SG2) as starting geometries (SGs). (Top) 3D geometries of the SGs and relative stability (in kcal/mol) with respect to the optimised geometry at the B3LYP/6-31++G(d,p) level of theory. The purple colour identifies atoms involved in the perturbed bonds; (middle, 4 panels) evolution of the system energy and relevant angles and dihedrals during the first 1 ps of the simulation; (bottom) 3D geometry of the system and relative energy compared to the nearest local minima after 1 ps of simulation time.

4. Effect of simulation temperature on the prediction of restoring forces

We deployed the MAL-1000-MF5 model in NVT simulations at 300 K, 500 K, 800 K and 1000 K to investigate the effect of the simulation temperature on the model's ability to relax a non-physical starting geometry (SG) through the prediction of restoring forces. The SG was generated by compressing the two C-C bonds, each to 0.9 Å, and stretching the two C=O bonds, each to 2.0 Å. The resulting geometry was 1,149 kcal/mol higher in energy than the optimised geometry of the system at the B3LYP/6-31++G(d,p) level of theory.

Figure **S5 (a-f)** illustrates the evolution of the system's energy (during the first 1 ps) and relevant bonds (during the first 10 ps) during the stabilisation phase. Figure **S5 (g-h)** depicts the maximum atomic force (`max-force`) during the same period. Note that `max-force` refers to the magnitude of the longest force vector acting on one of the atoms in the system. Notice that the system's energy (both total and potential energy) quickly drops at the beginning of the simulation. This period corresponds to the stabilisation phase, during which the model is trying to amend the initial bonds. Within 1000 steps, both C=O bonds are compressed from 2.0 Å to the more reasonable values of ~1.2 Å, while the C-C bonds are stretched from 0.9 Å to ~1.5 Å. Both the total and potential energies evolve in a wave-like manner, with peak positions shifted more to the right at lower temperatures. Furthermore, the bands on the total energy curve are more Gaussian-like than those on the potential energy curve. The reason for this observation is rooted in the statistical properties of the kinetic energy, the other component of the total energy. Indeed, unlike the potential energy, which fluctuates more dramatically due to various interactions that cause the vibration of bonds or rotation of angles, the kinetic energy is dictated by the system's temperature, which tends to fluctuate more smoothly following the Maxwell-Boltzmann distribution. Summing the kinetic and potential energies improves the smoothness of the total energy curve. Figure **S5(g)** suggests that the maximum atomic force goes from a huge restoring force of roughly 1 E_h/bohr to roughly 10^{-4} to 10^{-3} E_h/bohr at the end of the stabilisation phase. As anticipated, the higher the temperature, the higher the maximum atomic force at the end of the stabilisation phase.

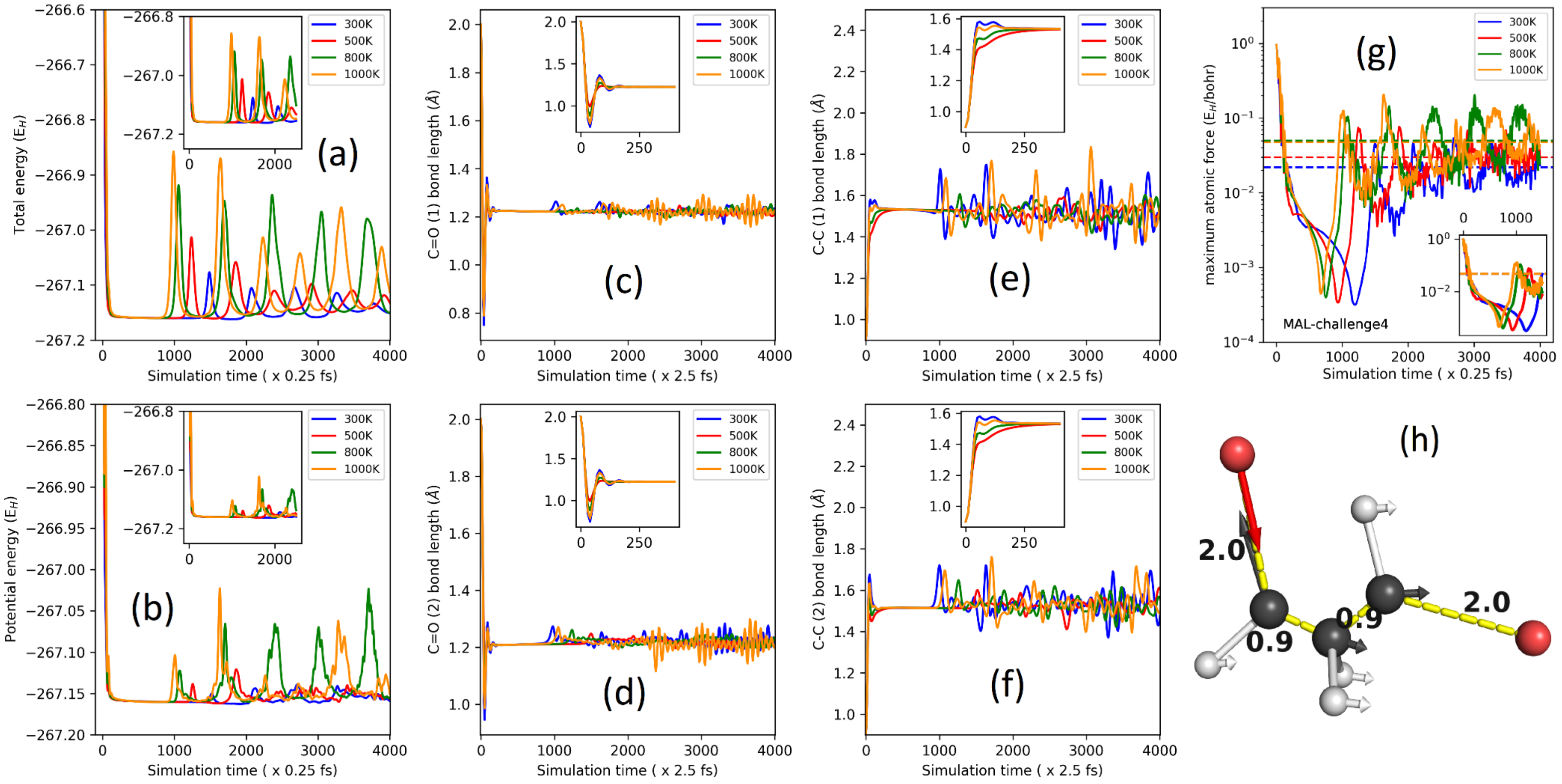

Figure S5. (a-b) Evolution of the MAL's total and potential energies during the first 4000 steps (1 ps); (c-f) Evolution of four bond lengths of interest during the first 40,000 steps (10 ps); (g) Maximum atomic force during the first 4000 steps (1 ps) of MD simulations initiated from six different high-energy geometries. The horizontal dashed lines indicate the average maximum force over the 4000 steps; (h) Starting geometry (SG) of the simulation. The bonds of interest are shown in yellow dashed lines. The force acting on the left C atom and that on the O atom, involved in the left C=O bond, are the largest and oriented (facing each other and thereby) compressing the bonds.

5. Optimisation of alanine dipeptide (AD) structures

Alanine dipeptide (AD) was chosen as a model system to assess the ability of FFLUX atomic energy models to reproduce the structure and potential energy of well-known stationary points. Three conformers of AD were considered, namely C5 (containing a 5-membered intramolecular hydrogen bond), C7ax and C7eq.

The starting geometries (SGs) were generated by randomly perturbing all Cartesian coordinates of the optimised structures of each conformer, with a maximum displacement of 0.25 Å. This procedure is considerably more aggressive than normal-mode distortions, in which atomic displacements are constrained along normal-mode vectors and typically result in only modest increases in potential energy. Figure **S6** and Tables **S2-S4** illustrate the structural and energetic diversity of these SGs. The most unstable SGs are 1615 kcal/mol (6757 kJ/mol), 2760 kcal/mol (11,548 kJ/mol) and 1980 kcal/mol (8284 kJ/mol) higher in energy compared to the nearest minima, namely C5, C7ax and C7eq, respectively.

As indicated in Figures **S6c–d**, the majority of the structural relaxation occurs within the first 20 optimisation steps, after which the FFLUX models reliably converge toward the corresponding minima. According to Table **S2**, FFLUX achieves a speedup of approximately two orders of magnitude (~ 200 times) relative to GAUSSIAN16. Moreover, the wall-clock times for FFLUX optimisations display a smaller standard deviation (Table **S3**), indicating a reduced sensitivity to the nature of the SG. In contrast, the potential energies and optimised geometries obtained with FFLUX exhibit slightly larger—yet still negligible—deviations compared to GAUSSIAN16. These discrepancies are attributed to increased force noise in the vicinity of the minima, where atomic forces become vanishingly small and difficult to predict correctly.

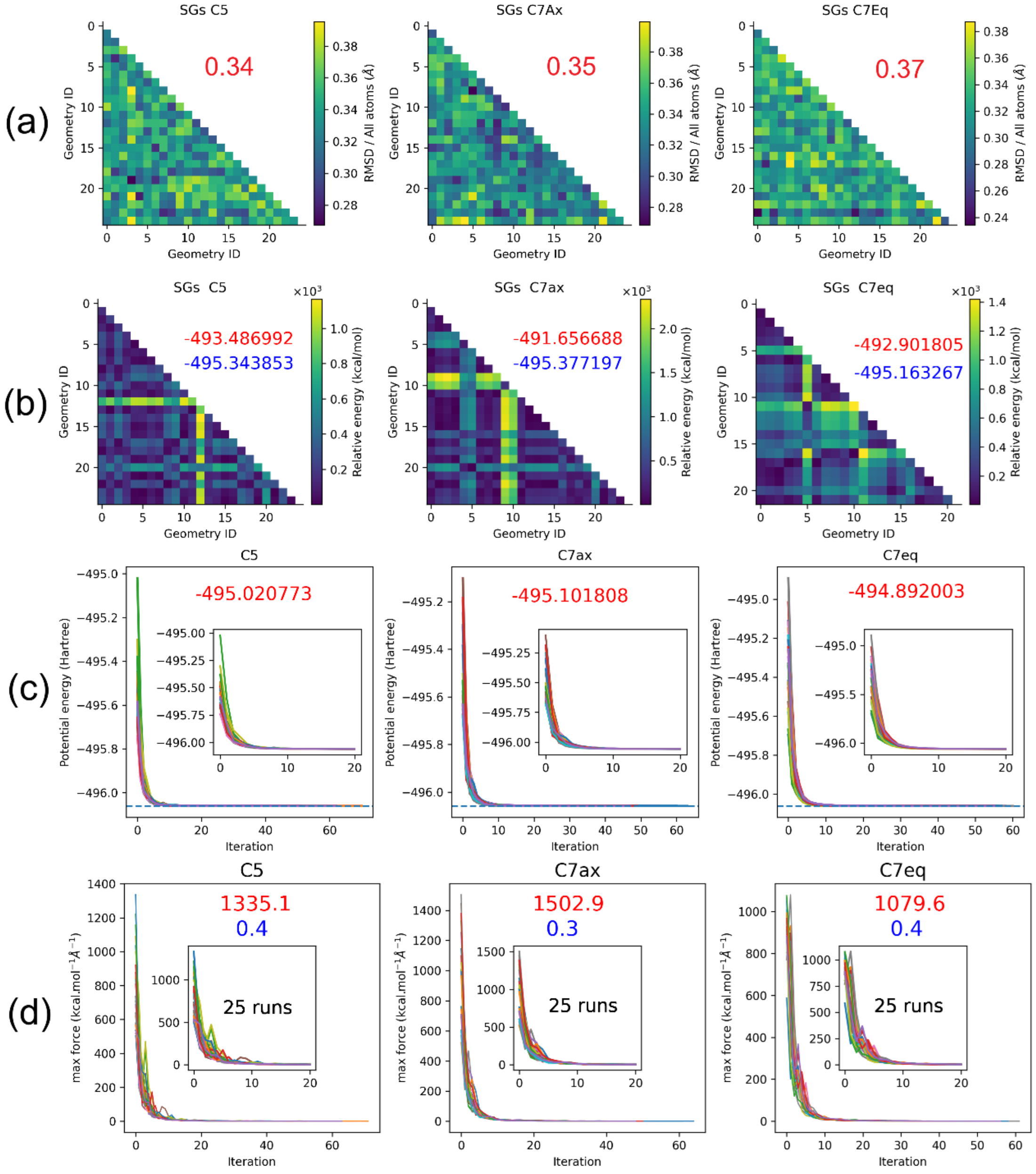

Figure S6. Structural and energetic diversity of AD SGs. The red number on the subplots (a) indicates the maximum RMSD with respect to the optimised structure of the corresponding conformer. On the subplots (b), the red and blue indicate the highest and lowest energy geometry among the SGs. Compared to the energies of the optimised structures, these structures are very high in energy. The most unstable (stable) among them are 1615 kcal/mol, 2760 kcal/mol and 1980 kcal/mol higher in energy compared to the nearest minima on the PES. (c)-(d) Optimisation paths showing the potential energy and maximum atomic forces as a function of the number of iterations completed. The number in red on subplots (c) is the FFLUX-estimated highest potential energy for each set of 25 geometries. Notice that this number is way off compared to the reference values in subplots (b). This is due to severe extrapolation. However, the models still predict restoring forces, pushing the system where they have more information and make reasonable prediction errors. The numbers in red and blue in the subplots (d) respectively indicate the maximum and minimum atomic force reached during the optimisation process.

Table S2. Computation cost of AD geometry optimisations using FFLUX models deployed in ASE (in comparison with GAUSSIAN16). All calculations were performed using 8 CPU cores and the same stopping condition to ensure a fair comparison of timings. We report average wall times in seconds. The numbers in parentheses are the standard deviations.

**Method**	**C5**	**C7ax**	**C7eq**
FFLUX	22.7	19.1	21.6
	(1.8)	(1.4)	(6.5)
GAUSSIAN16	4201.2	4528.0	4616.2
	(995.9)	(1312.6)	(1952.6)

Table S3. Optimised ϕ and ψ dihedral angles of AD conformers obtained using FFLUX and GAUSSIAN16. We report average values (in degrees) over 25 runs and the standard deviation in parentheses.

**Method**	**C5**	**C7ax**	**C7eq**
	ϕ		
FFLUX	-163.7	67.8	-81.7
	(1.26)	(0.77)	(1.11)
GAUSSIAN16	-167.7	64.4	-78.3
	(0.03)	(0.04)	(0.18)
	ψ		
FFLUX	172.3	-29.8	58.9
	(1.01)	(2.5)	(2.0)
GAUSSIAN16	167.3	-39.9	62.7
	(0.02)	(0.2)	(0.28)

Table S4. RMSD between FFLUX and GAUSSIAN16-optimised structures of AD conformers. RMSD values are expressed in Å.

**Atoms**	**C5**	**C7ax**	**C7eq**
All atoms	0.09	0.10	0.06
	(0.02)	(0.03)	(0.02)
Heavy atoms	0.06	0.07	0.03
	(0.01)	(0.01)	(0.01)

6. Diversity-aware selection (DAS) of geometries

The DAS approach relies on the assumption that the most representative subset is the one that preserves the structural diversity of an existing collection of geometries. Finding such a subset can be formulated as solving the following optimisation problem:

$$\hat{S} = arg \max_{\forall S \subset P} \left(\sum_{\forall G^* \in S} \Delta_{G^*}^{S} \right) \qquad (S1)$$

where argmax () returns the subset that maximises the sum $\sum_{\forall G^* \in S} \Delta_{G^*}^{S}$, P is a large collection of geometries (the parent population) and S is the most informative subset of P. The quantity $\Delta_{G^*}^{S}$ measures the dissimilarity between a test geometry G^* and a temporary subset $S_{temp} = S \backslash G^*$. This quantity is calculated as:

$$\Delta_{G^*}^{S} = \min_{y \in S_{temp}} \left(RMSD_{G^*}^{y} \right) \qquad (S2)$$

which implies that the similarity between test geometry G* and the excluded temporary subset S_{temp} is determined by the distance (in terms of RMSD) between the G^* and the most similar geometry found in S_{temp}.

An exact solution to the problem in Eq. S1 requires the enumeration of all possible combinations of |P| geometries taken |S| by |S|, i.e., $\binom{|P|}{|S|} = \frac{|P|!}{|S|!(|P|-|S|)!}$ possibilities, where "!" is the factorial operator and $|P|$ and $|S|$ are the cardinals (number of geometries) of the P and S datasets, respectively. However, such a naïve and brute force approach quickly becomes impractical due to a combinatorial explosion. Instead, our DAS implementation adopts a greedy approach similar to that in the work[11] of Yu and Kim.

The main idea is to populate the subset $\hat{S}$ by iteratively selecting hit geometries from the parent trajectory *P*. Note that $\hat{S}$ is the same as S (during the DAS process) and it only becomes $\hat{S}$ at the end of the iterative process) Unlike the previous approach[11], whose outcome fluctuates with the nature and the number of randomly selected seed geometries, the DAS procedure is semi-deterministic as it relies on a unique seed geometry. The latter is either user-supplied or defined as the nearest geometry to the centroid of the parent trajectory. Once the seed has been defined, the next hit geometry is chosen to coincide with the geometry with the largest shortest distance to every geometry already selected. This selection loop is repeated until a certain number of geometries have been selected (this number is predefined by the user and corresponds to the size of the subset $\hat{S}$). During the selection process, we make sure that selected geometries are removed from (a copy of) the parent trajectory. The pseudo-code of the DAS procedure is shown in **Algorithm 1**.

The similarity between geometries is evaluated by computing pairwise root mean square deviations (RMSD) between pre-aligned geometries. The alignment process relies on the well-known Kabsch-Umeyama algorithm to find the optimal rotation matrix between two geometries defined in Cartesian coordinates (the reference geometry is either provided by the user or chosen as the first geometry of the parent trajectory). For efficiency, the RMSD matrix is built beforehand, turning the selection loop into a mere lookup task. Insufficient memory issues are prevented by constructing the RMSD matrix (which has size N_{geom} x N_{geom}) in horizontal blocks. Each block has a maximum size of $N_{geom} \times c$ where N_{geom} is the number of geometries in *P*, and c is a user-defined chunk size (default value 250).

Algorithm 1: Pseudo-code of the DAS procedure

Input: parent population (P), chunk size (c), and sample size ($|\hat{S}|$)
Output: most diverse subset $\hat{S}$
Read the XYZ trajectory containing the parent population
Specify seed geometry (s0)
Rotate geometries and seed geometry with respect to frame0
$P_{temp} \leftarrow P$
$\hat{S}_{temp} \leftarrow$ most similar geometry to the seed geometry (s0)
$P_{temp} \leftarrow P_{temp} -$ s0
$N = 0$
while $N \leq |\hat{S}|$-1 **do**
    Find the next hit geometry (h*) by solving Eq. S1
    $P_{temp} \leftarrow P_{temp} -$ h*
    $\hat{S}_{temp} \leftarrow \hat{S}_{temp} +$ h*
    $N \leftarrow N + 1$
done

References

1. Nahum, O. E., Yosipof, A. & Senderowitz, H. A multi-objective genetic algorithm for outlier removal. *J. Chem. Inf. Model.* **55**, 2507–2518 (2015).

2. Mellenbergh, G. J. Outliers. In *Counteracting Methodological Errors in Behavioral Research*, 293–308 (Springer Nature Switzerland, Cham, 2019).

3. Popelier, P. L. A. A method to integrate an atom in a molecule without explicit representation of the interatomic surface. *Comput. Phys. Commun.* **108**, 180–190 (1998).

4. Aicken, F. M. & Popelier, P. L. A. Atomic properties of selected biomolecules. Part 1. The interpretation of atomic integration errors. *Can. J. Chem.* **78**, 415–426 (2000).

5. Matta, C. F. & Boyd, R. J. An introduction to the quantum theory of atoms in molecules. In *The Quantum Theory of Atoms in Molecules,* **1** (Wiley-VCH Verlag GmbH & Co. KGaA, Weinheim, 2007).

6. Buemi, G. & Gandolfo, C. Malondialdehyde and acetylacetone. An AM1 study of their molecular structures and keto–enol tautomerism. *J. Chem. Soc., Faraday Trans. 2: Mol. Chem. Phys.* **85**, 215–227 (1989).

7. Buemi, G. & Zuccarello, F. Ab initio study of the potential-energy well of malondialdehyde on varying the O···O distance. *J. Chem. Soc., Faraday Trans.* **92**, 347–351 (1996).

8. Cersonsky, R. K., Helfrecht, B. A., Engel, E. A., Kliavinek, S. & Ceriotti, M. Improving sample and feature selection with principal covariates regression. *Mach. Learn.: Sci. and Technol.* **2**, 035038 (2021).

9. Wang, J., Wolf, R. M., Caldwell, J. W., Kollman, P. A. & Case, D. A. Development and testing of a general amber force field. *J. Comput. Chem.* **25**, 1157–1174 (2004).

10. Bannwarth, C., Ehlert, S. & Grimme, S. GFN2-xTB—An accurate and broadly parametrized self-consistent tight-binding quantum chemical method with multipole electrostatics and density-dependent dispersion contributions. *J. Chem. Theory Comput.* **15**, 1652–1671 (2019).

11. Yu, H. & Kim, S. Passive sampling for regression. In *2010 IEEE International Conference on Data Mining*. IEEE (2010).
